# Affective and cognitive theory of mind and associated brain functional alterations in frontotemporal dementia

**DOI:** 10.1093/braincomms/fcag216

**Published:** 2026-06-06

**Authors:** Chiara Tripodi, Elisa Canu, Anna Marangon, Veronica Castelnovo, Silvia Basaia, Edoardo G Spinelli, Giordano Cecchetti, Alma Ghirelli, Stefano Pisano, Giulia Rugarli, Francesca Caso, Giuseppe Magnani, Paola Caroppo, Sara Prioni, Cristina Villa, Lucio Tremolizzo, Ildebrando Appollonio, Federico Verde, Nicola Ticozzi, Vincenzo Silani, Massimo Filippi, Federica Agosta

**Affiliations:** Neuroimaging Research Unit, Division of Neuroscience, IRCCS San Raffaele Scientific Institute, Milan 20132, Italy; Neurology Unit, IRCCS San Raffaele Scientific Institute, Milan 20132, Italy; Vita-Salute San Raffaele University, Milan 20132, Italy; Neuroimaging Research Unit, Division of Neuroscience, IRCCS San Raffaele Scientific Institute, Milan 20132, Italy; Neurology Unit, IRCCS San Raffaele Scientific Institute, Milan 20132, Italy; Neuroimaging Research Unit, Division of Neuroscience, IRCCS San Raffaele Scientific Institute, Milan 20132, Italy; Vita-Salute San Raffaele University, Milan 20132, Italy; Neuroimaging Research Unit, Division of Neuroscience, IRCCS San Raffaele Scientific Institute, Milan 20132, Italy; Neurology Unit, IRCCS San Raffaele Scientific Institute, Milan 20132, Italy; Neuroimaging Research Unit, Division of Neuroscience, IRCCS San Raffaele Scientific Institute, Milan 20132, Italy; Neuroimaging Research Unit, Division of Neuroscience, IRCCS San Raffaele Scientific Institute, Milan 20132, Italy; Neurology Unit, IRCCS San Raffaele Scientific Institute, Milan 20132, Italy; Vita-Salute San Raffaele University, Milan 20132, Italy; Neuroimaging Research Unit, Division of Neuroscience, IRCCS San Raffaele Scientific Institute, Milan 20132, Italy; Neurology Unit, IRCCS San Raffaele Scientific Institute, Milan 20132, Italy; Neuroimaging Research Unit, Division of Neuroscience, IRCCS San Raffaele Scientific Institute, Milan 20132, Italy; Neurology Unit, IRCCS San Raffaele Scientific Institute, Milan 20132, Italy; Vita-Salute San Raffaele University, Milan 20132, Italy; Neuroimaging Research Unit, Division of Neuroscience, IRCCS San Raffaele Scientific Institute, Milan 20132, Italy; Neurology Unit, IRCCS San Raffaele Scientific Institute, Milan 20132, Italy; Neuroimaging Research Unit, Division of Neuroscience, IRCCS San Raffaele Scientific Institute, Milan 20132, Italy; Neurology Unit, IRCCS San Raffaele Scientific Institute, Milan 20132, Italy; Vita-Salute San Raffaele University, Milan 20132, Italy; Neurology Unit, IRCCS San Raffaele Scientific Institute, Milan 20132, Italy; Neurology Unit, IRCCS San Raffaele Scientific Institute, Milan 20132, Italy; Fondazione IRCCS Istituto Neurologico Carlo Besta, Unit of Neurology 5—Neuropathology, Milan 20133, Italy; Fondazione IRCCS Istituto Neurologico Carlo Besta, Unit of Neurology 5—Neuropathology, Milan 20133, Italy; Fondazione IRCCS Istituto Neurologico Carlo Besta, Unit of Neurology 5—Neuropathology, Milan 20133, Italy; Neurology Unit, ‘San Gerardo’ Hospital and University of Milano-Bicocca, Monza 20900, Italy; Neurology Unit, ‘San Gerardo’ Hospital and University of Milano-Bicocca, Monza 20900, Italy; Department of Neurology and Laboratory of Neuroscience, IRCCS Istituto Auxologico Italiano, Milan 20149, Italy; Department of Neurology and Laboratory of Neuroscience, IRCCS Istituto Auxologico Italiano, Milan 20149, Italy; ‘Dino Ferrari’ Center, Department of Pathophysiology and Transplantation, Università Degli Studi di Milano, Milan 20122, Italy; Department of Neurology and Laboratory of Neuroscience, IRCCS Istituto Auxologico Italiano, Milan 20149, Italy; ‘Dino Ferrari’ Center, Department of Pathophysiology and Transplantation, Università Degli Studi di Milano, Milan 20122, Italy; Neuroimaging Research Unit, Division of Neuroscience, IRCCS San Raffaele Scientific Institute, Milan 20132, Italy; Neurology Unit, IRCCS San Raffaele Scientific Institute, Milan 20132, Italy; Vita-Salute San Raffaele University, Milan 20132, Italy; Neurorehabilitation Unit, IRCCS San Raffaele Scientific Institute, Milan 20132, Italy; Neurophysiology Service, IRCCS San Raffaele Scientific Institute, Milan 20132, Italy; Neuroimaging Research Unit, Division of Neuroscience, IRCCS San Raffaele Scientific Institute, Milan 20132, Italy; Neurology Unit, IRCCS San Raffaele Scientific Institute, Milan 20132, Italy; Vita-Salute San Raffaele University, Milan 20132, Italy

**Keywords:** social cognition, theory of mind (ToM), frontotemporal dementia (FTD), functional connectivity

## Abstract

Theory of mind (ToM), the ability to infer others’ beliefs (cognitive ToM) and emotions (affective ToM), is compromised in behavioural variant frontotemporal dementia (bvFTD). However, its diagnostic and prognostic value in other frontotemporal dementia (FTD) variants remains underexplored due to limited understanding of the underlying neural mechanisms. This study investigated whether ToM deficits are shared across the frontotemporal dementia spectrum and explored the functional connectivity alterations underlying these disturbances using resting-state functional magnetic resonance imaging. Sixty-seven FTD patients [14 non-fluent variant primary progressive aphasia (nfvPPA), 17 semantic variant primary progressive aphasia (svPPA), 23 bvFTD, 13 right temporal variant frontotemporal dementia (rtvFTD); 34 women; mean age 66.5 ± 7.7 years] and two control groups (48 age-matched healthy controls; 50 young healthy controls) underwent clinical, neuropsychological and brain magnetic resonance imaging assessments. ToM was evaluated in patients using the Story-Based Empathy Task (SET), which includes the Story-Based Empathy Task affective subtest (SET-EA) and the Story-Based Empathy Task cognitive subtest (SET-IA). Resting-state functional connectivity networks were obtained in young healthy controls using seed-based analysis centred on the left medial prefrontal cortex for affective ToM and the right supramarginal gyrus for cognitive ToM. In addition, four large-scale functional networks were reconstructed to reflect disease-specific vulnerability. Functional brain connectivity within all networks was quantified using graph analysis and connectomics, and between-group comparisons were performed on both global and seed-based regional metrics. All patient groups showed similar impairments in affective and cognitive ToM performance. Network analyses revealed two dissociable but interconnected ToM systems. Global metrics of network topology indicated increased path length and reduced nodal strength in both ToM networks, particularly in bvFTD and nfvPPA patients (*P* < 0.05). Direct seed-based connectivity analyses confirmed widespread functional connectivity reductions from key nodes (e.g. left inferior frontal gyrus, anterior cingulate cortex) in these groups. In contrast, svPPA and rtvFTD cases exhibited relatively preserved functional connectivity within ToM circuits. Correlation analyses revealed associations between cognitive ToM network metrics and global ToM performance, and between functional connectivity in the salience network and behavioural dysfunction. Affective and cognitive ToM abilities are comparably impaired across FTD variants, suggesting that socio-cognitive impairments may represent a core and early feature across the FTD spectrum. Such deficits are mirrored by patterns of functional disconnection within dedicated large-scale networks, with bvFTD and nfvPPA showing the most pronounced disruptions. This study underscores the diagnostic relevance of socio-cognitive markers and highlights their potential as clinical and biomarker targets in future therapeutic interventions.

## Teaser

Tripodi *et al*. report that patients with different variants of frontotemporal dementia share difficulties in understanding others’ thoughts and feelings, caused by disruptions in social brain networks. Impairments are most pronounced in behavioural frontotemporal dementia and non-fluent variant primary progressive aphasia and may inform diagnosis and guide interventions.

## Graphical Abstract

**Graphical Abstract:**
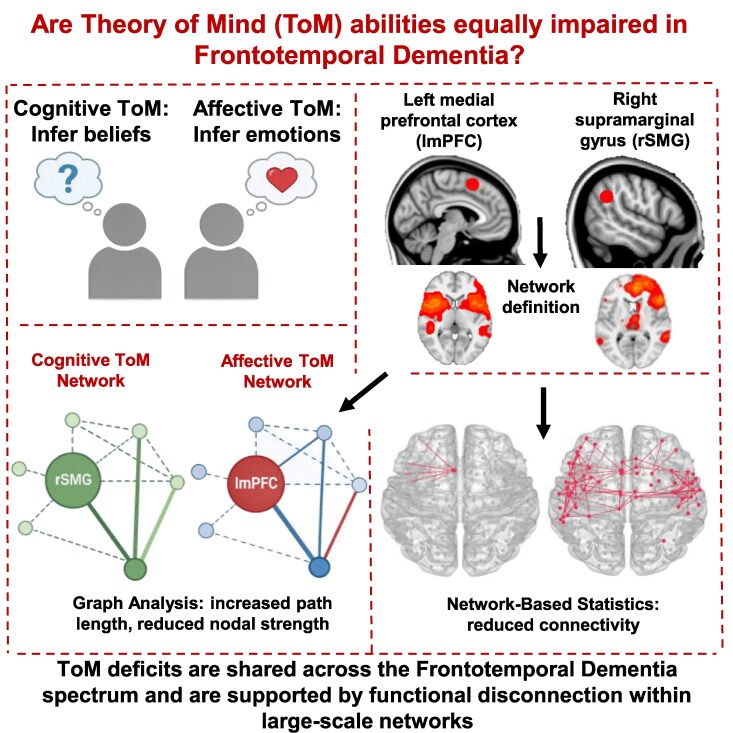

## Introduction

To actively take part in the social world, human beings require a neurocognitive process that goes beyond perception, in order to decode others’ mental states and shape their judgments, decisions and behaviours subsequently.^1^ Among social cognition sub-domains, theory of mind (ToM) refers to the ability to infer others’ beliefs (cognitive ToM) and to understand others’ emotional states (affective ToM).^2^

Up to now, affective and cognitive ToM skills have been amply investigated in the behavioural variant of frontotemporal dementia (bvFTD), whose diagnostic criteria are anchored in conduct disturbances.^3^ Within the FTD spectrum, neurodegeneration targeting the right anterior temporal lobe (rATL) is often associated with subtle language alterations and behavioural disturbances.^4^ This phenotype is referred to as right temporal variant FTD (rtvFTD), and its behavioural manifestations include scarce empathic concern as well as altered ToM abilities.^5^

Over recent years, a growing body of research has highlighted significant socioemotional disorders across primary progressive aphasia (PPA) syndromes,^6^ including the semantic (svPPA) and non-fluent (nfvPPA) variants, which also belong to the FTD spectrum.^7^ Investigating whether ToM alterations represent core symptoms of FTD rather than a consequence of disease severity carries critical diagnostic implications. However, extra-linguistic features characterizing PPA syndromes are often underestimated or neglected, due to the depth level reached by the assessment. Furthermore, studies evaluating social-cognitive capacities in PPA patients primarily employ verbal tasks, which makes it difficult to determine whether pathological scores are due to scarce socio-emotional skills or poor understanding of the test.

Recent studies explored the activation patterns for cognitive as opposed to affective ToM, suggesting how the two components of ToM are supported by discrete but interdependent neural systems.^8^ More precisely, affective ToM appears to be associated with a more anterior and ventral network, centred on the medial prefrontal cortex (mPFC) and encompassing the inferior frontal gyrus (IFG) and bilateral orbitofrontal cortex (OFC). On the other hand, cognitive ToM seems to relate to the activity of more posterior regions, including the supramarginal gyrus (SMG), inferior parietal lobe (IPL), precuneus and the dorsolateral prefrontal cortex.^9-13^ Still, no definitive consensus has been reached as regards the neural networks that support mentalizing skills. Broadening our understanding of the functional circuitry responsible for ToM abilities in the general population may provide a model to evaluate the integrity of such networks in a clinical scenario.

The present study aimed at investigating cognitive and affective ToM abilities in svPPA and nfvPPA syndromes, as well as at comparing these skills with other FTD variants, such as bvFTD and rtvFTD cases. To do so, patients underwent a comprehensive cognitive evaluation, which included the Story-Based Empathy Task (SET),^14^ a neuropsychological assessment that investigates both affective and cognitive ToM abilities without the use of verbal stimuli.^15^ Moreover, this study explored the distinctive neural correlates of affective and cognitive ToM alterations in patients. Through a seed-based resting-state functional connectivity (rs-FC) approach, we reconstructed two ToM brain networks using left mPFC (lmPFC) and right SMG (rSMG) as seeds for the affective and cognitive ToM circuits, respectively. Furthermore, global and regional rs-FC within the two ToM networks were assessed with graph analysis and connectomics.

To provide converging evidence for our findings, we examined connectivity within four large-scale brain networks selected to be representative of the core neuroanatomical alterations typically associated with each FTD subtype. Specifically, the following functional networks were assessed: left anterior temporal (left ATL) and right anterior temporal lobe (right ATL) networks for svPPA and rtvFTD phenotypes, respectively; left inferior frontal gyrus (left IFG) network for nfvPPA cases; and the salience network (SN) centred on the right anterior cingulate cortex for the bvFTD.

We expected to find similarly impaired affective and cognitive ToM skills in all patient groups, including PPA, and to observe disrupted interregional functional coupling and network-specific connectivity profiles consistent with the underlying syndromes.

## Materials and methods

### Subjects

A total of 292 patients with a suspected diagnosis of FTD-related disorders were prospectively enrolled at five referral clinics in Lombardy, Italy, and referred to the IRCCS San Raffaele Scientific Institute in Milan between May 2017 and July 2023 to perform MRI on a 3T scanner, as part of their diagnostic work-up. Among them, we selected patients who received a clinical diagnosis of one of the following clinical variants (i.e. bvFTD,^3^ rtvFTD,^4^ nfvPPA and svPPA^7^); performed clinical assessment, neuropsychological battery including an evaluation of affective and cognitive ToM with the subtests of the SET; and brain structural and resting-state functional MRI (rs-fMRI) (see details below). All patients received a diagnosis of probable FTD according to current consensus criteria,^3,4,7^ following a comprehensive multidisciplinary evaluation involving neurologists, neuropsychologists and neuroradiologists. Exclusion criteria were: significant medical illnesses or substance abuse that could interfere with cognitive functioning; any (other) major systemic, psychiatric or neurological illnesses; lacunae and extensive cerebrovascular disorders at MRI; artefacts at MRI.

The final cohort included 67 sporadic FTD cases (23 bvFTD, 13 rtvFTD, 14 nfvPPA, 17 svPPA). Thirty-two patients (14 bvFTD, 8 rtvFTD, 4 nfvPPA and 6 svPPA) also underwent a lumbar puncture to exclude cerebrospinal fluid biomarker profiles suggestive of Alzheimer’s disease pathology, as part of their diagnostic work-up.

For the MRI analysis, two independent groups of healthy controls were retrospectively selected among those recruited by the Neuroimaging Research Unit, IRCCS San Raffaele Scientific Institute, between 2017 and 2018. The first group consisted of 48 age- and education-matched healthy controls (HC), who underwent MRI assessment and a completed neuropsychological evaluation. The second group consisted of 50 young healthy controls [HC-Y, age: 25.25 ± 2.75 years; 23 (46%) women; education: 15.5 ± 2.7 years], recruited among students at the Vita-Salute San Raffaele University in Milan, selected for the construction of brain rs-fMRI networks. All controls were recruited based on the following criteria: no family history of neurodegenerative diseases, and normal neurological and cognitive assessment.

Exclusion criteria for all subjects were: medical illnesses or substance abuse that could interfere with cognitive functioning; any (other) major systemic, psychiatric or neurological illnesses; and other causes of focal or diffuse brain damage, including lacunae and extensive cerebrovascular disorders at routine MRI.

The local ethical standards committee on human experimentation approved the study protocol and all participants provided written informed consent prior to study inclusion, according to the Declaration of Helsinki.

### Clinical evaluation

Clinical evaluations were performed by experienced neurologists blinded to MRI results. For all patients, disease severity was assessed using the FTLD-Clinical Dementia Rating Scale (FTLD-CDR)^16^ and independence with basic (ADL)^17^ and instrumental activities of daily life (IADL).^18^ The FTLD-CDR was scored following the procedure described by the Mayo Clinic^19^ and subsequently adapted into Italian with the contribution of Borroni *et al*. (2010). The sum-of-boxes approach was used, encompassing the six standard CDR domains as well as the two FTLD-specific domains (Language and Behaviour/Comportment). Accordingly, higher FTLD-CDR scores reflect greater disease severity.

### Neuropsychological assessment

Neuropsychological assessments were performed by experienced neuropsychologists. In all patients, ToM was evaluated using the SET, a non-verbal battery in which subjects are asked to predict a series of events by selecting graphic vignettes. The test is composed of 18 stimuli, divided into two experimental conditions that allow for the assessment of one’s capacity to infer other’s intentions (SET-IA) and emotions (SET-EA), and one control condition (SET-CI) that evaluates one’s capacity to infer causality. Scores in each SET subtest were calculated for each patient according to Italian normative data,^14^ and three scores were considered for the analyses: the IA score, the EA score and the global score. No exclusion criteria were applied based on SET-CI performance, but the control condition was not included in the main correlational analyses, which focused on ToM-specific components in line with the study aims.

The following cognitive functions were also investigated: global cognitive functioning with the Mini-Mental State Examination (MMSE)^20^ and the Frontal Assessment Battery (FAB);^21^ long- and short-term verbal memory with the Rey Auditory Verbal Learning Test^22^ and the digit span forward,^23^ respectively; long- and short-term spatial memory with the recall and recognition of the Benson’s complex figure^24^ and the spatial span forward;^23^ abstract reasoning with the Raven’s Coloured Progressive Matrices (RCPM);^25^ executive functions with the digit span backward,^26^ the Clock drawing test (CDT),^27^ the Attentive Matrices,^28^ the Trail Making Test (TMT)^29^ and the Modified Card Sorting Test;^29^ language with the Token test,^30^ and phonemic fluency tests;^31^ visuospatial abilities with the copy of the Benson’s complex figure,^24^ and with the freehand copy of drawings with and without landmarks;^22^ emotion recognition with the abbreviated version of the Comprehensive Affect Testing System (CATS-A).^32^ The presence of behavioural disturbances was assessed with the Neuropsychiatric Inventory (NPI),^33^ and the Frontal Behavioural Inventory (FBI)^34^ administered to patients’ caregivers.

HC underwent the same assessment of patients except for FAB and SET. Moreover, in HC, the Beck Depression Inventory (BDI)^35^ was used to exclude subjects with mood alterations.

### MRI acquisition

All participants underwent brain MRI on a 3.0 T scanner (Ingenia CX, Philips), as previously described.^36^ The following brain MRI sequences were obtained from all participants: 3D T1-weighted (TFE) (TR = 7 ms; TE = 3.2 ms; flip angle = 9°; 204 contiguous sagittal slices with voxel size = 1 × 1 × 1 mm, matrix size = 256 × 240, FOV = 256 × 240 mm^2^); 3D FLAIR (TR = 4800 ms; TE = 267 ms; TI = 1650 ms; ETL = 167; NEX = 2; 192 contiguous sagittal slices with voxel size = 0.89 × 0.89 × 1 mm, matrix size = 256 × 256, FOV = 256 × 256 mm^2^); 3D T2 (TR = 2500 ms; TE = 330 ms; ETL = 117; NEX = 1; 192 contiguous sagittal slices with voxel size = 0.89 × 0.89 × 1 mm, matrix size = 256 × 258, FOV = 256 × 256 mm^2^); and T2* weighted (GE-EPI) sequence for rs-fMRI (TR = 1567 ms; TE = 35 ms; flip angle = 70°; MB = 2; SENSE = 2; FOV = 240 × 240; pixel size = 2.5 × 2.5 mm; slice thickness=3 mm; 320 sets of 48 contiguous axial slices; acquisition time = 8′ and 32″). Before starting the rs-fMRI scanning, the technician talked with the participants through their earphones instructing them to remain motionless, to keep their eyes closed, not to fall asleep and not to think about anything in particular.

### MRI analysis

MRI analysis was performed by experienced observers, blinded to the identity of subjects.

#### RS fMRI preprocessing

Resting-state fMRI data were processed using the FMRIB Software Library (FSL, version 5.0), following previously described procedures.^37^ The first four volumes of the rs-fMRI data were removed to reach complete magnet signal stabilization. The following FSL-standard preprocessing pipeline was implemented: (i) motion correction using MCFLIRT; (ii) high-pass temporal filtering (lower frequency: 0.01 Hz); (iii) spatial smoothing (Gaussian kernel of FWHM 6 mm); (iv) single-session independent component analysis-based automatic removal of motion artefacts (ICA_AROMA) in order to identify those independent components (ICs) representing motion-related artefacts.

#### Seed-based network definition in young healthy subjects

**Figure 1:**
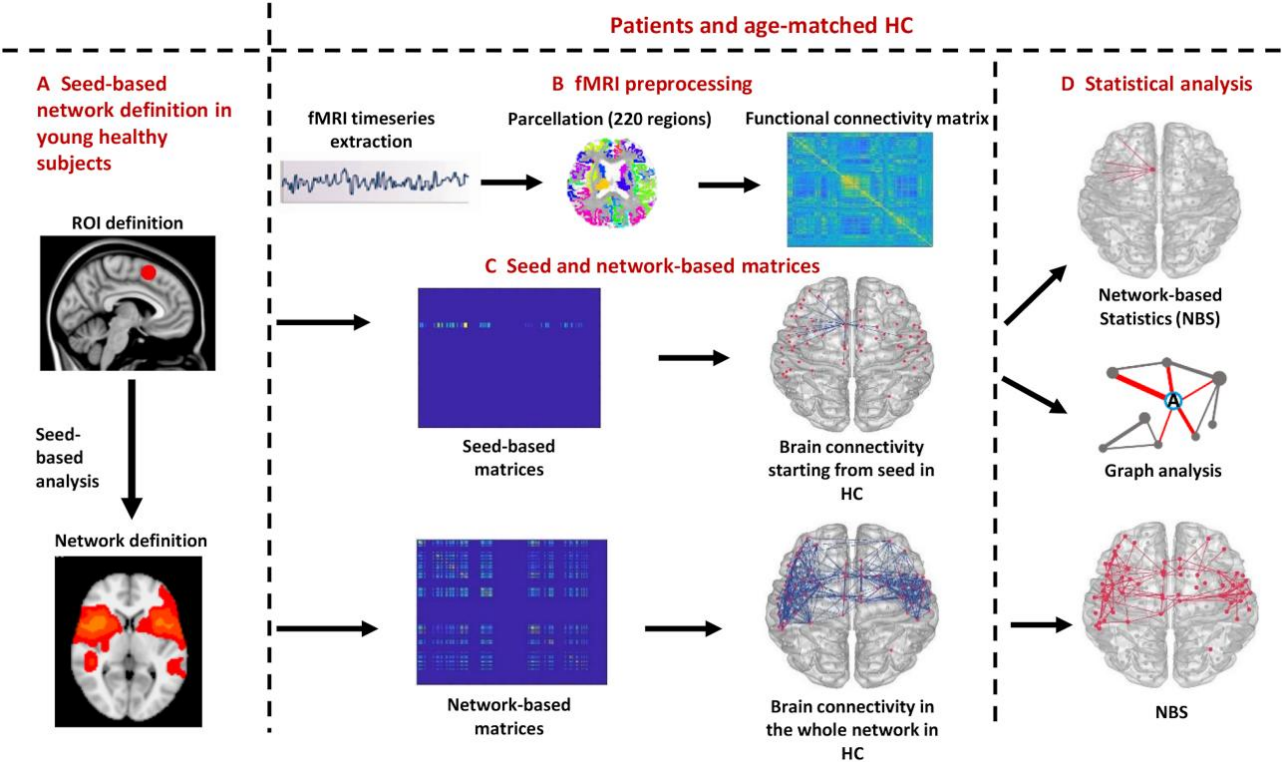
**Graphical representation of methods.** (**A**) Seed-based network definition in young healthy individuals. A region of interest (ROI) was selected and used to derive a seed-based functional connectivity map for subsequent network definition. (**B**) fMRI preprocessing in patients and age-matched healthy controls (HC), including grey matter parcellation into 220 regions, time series extraction and computation of individual functional connectivity matrices. (**C**) Construction of seed-based and network-based connectivity matrices, followed by the estimation of subject-specific brain connectivity patterns starting from the seed or from the entire network. (**D**) Statistical analysis. Group comparisons were performed using network-based statistics (NBS) and graph-theoretical measures at network and seed level.

We first obtained affective and cognitive ToM, as well as language, salience and anterior temporal network maps by applying a seed-based analysis to the rs-fMRI data of HC-Y, following the procedure described in Cecchetti *et al*.^38^ (Fig. 1, section A). Based on a recent meta-analysis on healthy middle-aged adults,^10^ two regions of interest (ROIs) were created as main nodes for affective and cognitive ToM, respectively: lmPFC (10-mm radius sphere centred at the MNI coordinates −2, 13, 55) and rSMG (10-mm radius sphere centred at the MNI coordinates 61, −49, 30). In HC-Y, a seed-based rs-FC analysis was performed between the two nodes, separately, and the rest of the brain. Two further ROIs were created as main nodes of the language networks based on previous knowledge:^39^ the pars triangularis of the left inferior frontal gyrus (IFG, 10-mm radius sphere centred at the MNI coordinates −54,24,3) and the lateral portion of the left anterior temporal lobe (lATL, 10-mm radius sphere centred at the MNI coordinates −50,11, −32). Due to the lack of evidence concerning the localization of the right-lateralized ATL network, MNI coordinates mirroring values used for the left ATL network were selected (rATL, 10-mm radius sphere centred at the MNI coordinates 50, 11, −32). A node for the salience network (SN, 10-mm radius sphere centred at the MNI coordinates 6, 30, 28, corresponding to the anterior cingulate cortex) was also created based on previous literature.^39^ All seeds were defined in the MNI space and moved to each subject’s native T1-weighted space through non-linear and affine registrations, and visually inspected in the individual brains by neuroimaging experts. Seed-based rs-fMRI networks were then obtained using a two-step regression analysis as implemented in the FMRIB software library (FSLv5). First, time series of white matter, cerebrospinal fluid and whole brain volumes in rs-fMRI native space were extracted from the preprocessed and denoised data, and their effects were regressed out using the FMRI Expert Analysis Tool. Seed mean time series were then calculated. The output of this step is represented by subject-level maps of all positively and negatively predicted voxels for each regressor. Subject-level maps were registered to the MNI standard template to enter the statistical analysis. rs-fMRI networks mean connectivity maps between each voxel and the rest of the brain were created using GLM, which included fMRI networks maps as dependent variables. Corrections for multiple comparisons were carried out at a cluster level using Gaussian random field theory, *z* > 2.3; cluster significance: *P* < 0.05, corrected for multiple comparisons.

#### Connectome in FTD patients and age-matched HC

Connectome reconstruction was performed as described in Filippi *et al*.^40^ In FTD patients and age-matched HC, grey matter (GM) from T1-weighted image was parcellated into 220 similarly sized brain ROIs, which included the cerebral cortex and basal ganglia but excluded the cerebellum.^40^ Cortical GM was segmented using SPM12, while basal ganglia (i.e. bilateral caudate, globus pallidus, putamen and thalamus), hippocampus and amygdala maps were obtained using FIRST in FSL. In these subjects, FC matrices were obtained on the basis of correlation analysis (Fig. 1, section B). Mean time series were extracted from each of the 220 ROI by averaging the signal from all voxels within each region. rs-fMRI data were masked with the subject’s GM map in order to consider only voxels corresponding to GM and avoid the effect of atrophy. The Pearson’s correlation coefficient between the mean time series of each ROI pair, indicating the level of FC between regions *i* and *j*, was enter into cell *c*(*i*, *j*) of the matrix. Pearson’s correlation coefficients were then converted to *z*-scores using Fisher’s *r*-to-*z* transformation. Negative correlation coefficients were set to ‘not a number’ to mark these brain regions as unconnected.^41^ An undirected, weighted graph describing the brain’s network functional layout was obtained by computing correlations between the 220 GM ROIs. Functional connections were required to be present in a structural connectivity matrix of the 50 HC-Y, who represented a ‘reference’ healthy connectome, as previously reported.^42^

##### Seed and network-based matrices

To characterize the rs-fMRI-derived networks in FTD and age-matched HC, the network masks obtained from the seed-based FC analyses in HC-Y were binarized and resliced to match the spatial resolution and dimensions of the Automated Anatomical Labeling (AAL) atlas (220-region version). The binarized network mask was overlaid onto the AAL atlas, and voxels within the mask were assigned to their corresponding anatomical regions. For each network, only those AAL regions with more than 50% of their volume included in the network mask were retained. This procedure yielded a list of anatomical regions for each rs-fMRI-derived network that precisely defined its spatial extent.

For each subject (FTD patients and age-matched HC), we used the network-specific ROI lists to subset the whole-brain FC matrix (Fig. 1, section C). For each network, we selected all rows corresponding to its constituent ROIs, yielding a *network-based* connectivity matrix that contained only connections between regions within that network.

In a second step (Fig. 1, section C), we focused on the seed region of each network (identical to the seed used in the HC-Y analyses). For each subject and network, we extracted the single row corresponding to the seed and considered only the cells of the matrix linking the seed to the ROIs belonging to that same network, thus obtaining *seed-based* matrices (1 × N) that captured the direct connectivity from the seed to all other nodes in the network.

### Statistical analysis

#### Demographic, clinical and cognitive data

Data distribution was assessed using the Shapiro–Wilk test. Sociodemographic data were compared across groups (bvFTD, nfvPPA, svPPA, rtvFTD) using one-way ANOVA models, followed by Bonferroni *post hoc* correction for multiple comparisons, and Fisher’s exact test for continuous and categorical variables, respectively. Differences in clinical and neuropsychological data between groups were assessed using age-, sex- and education-adjusted ANOVA models followed by Bonferroni-corrected *post hoc* pairwise comparisons (*P* < 0.05) on rank-transformed data using SPSS software (SPSS Inc., Chicago, IL, version 24). According to Italian normative data, pathological scores in each SET subtest (IA, EA, CI and global score) were computed for each patient, and the distribution of pathological raw scores was compared across groups using Fisher’s exact test.

#### Network analysis

##### Global network analysis

Global properties of brain functional networks were explored using the Brain Connectivity MATLAB toolbox (brain-connectivity-toolbox.net). Graph-theoretical metrics, including nodal strength, characteristic path length, local efficiency and clustering coefficient, were assessed to characterize the topologic organization of global brain networks in FTD patients and healthy controls. Global network analysis was computed on the seed-based matrices to quantify the topology of seed-centred connectivity (Fig. 1, section D). Global functional metrics were tested for normality distribution using the Shapiro–Wilk test and compared between groups using age-, sex- and education-adjusted ANOVA models followed by Bonferroni-corrected *post hoc* pairwise comparisons (*P* < 0.05) on rank-transformed data using SPSS software (SPSS Inc., Chicago, IL, version 24).

##### Connectivity analysis

Network-based statistics (NBS)^43^ was performed to assess FC between each pair of nodes at a level of significance *P* < 0.05, performing all possible combinations of comparisons between study groups. For each contrast, a corrected *P*-value was calculated by means of a permutation analysis (5000 permutations) adjusted for age, sex and education levels. NBS was applied to both network-based and seed-based matrices to identify altered functional components at network and seed level (Fig. 1, section D).

##### Correlation between functional connectivity and neuropsychological data

In all patients, Spearman correlation analyses were conducted to examine the associations between clinical and cognitive measures and both global network metrics and mean FC. Metrics from the network’s seed were considered (see ‘Seed and network-based matrices’ for details). Correlation analyses were performed using raw test scores. Results were Bonferroni-corrected for multiple comparisons (*P* < 0.05). Specifically, correlations were tested with the FBI scale, the Token test and the SET (IA, EA and global score). No covariates (age, sex, education) were included in the correlation analyses.

## Results

### Demographic, clinical and cognitive data

Two hundred ninety-two patients were initially recruited. After exclusion of subjects according to exclusion criteria, 67 sporadic FTD cases (23 bvFTD, 13 rtvFTD, 14 nfvPPA, 17 svPPA) were included. Forty-eight age-matched HC and 50 HC-Y were also included according to the inclusion criteria. Sociodemographic and clinical characteristics of patients and HC stratified according to the clinical diagnosis are summarized in Table 1. Subjects were comparable in terms of age and education; bvFTD and rtvFTD groups differed from controls in terms of sex owning a greater number of males. All patients showed lower global cognition (i.e. MMSE) as compared to healthy subjects. There were no significant differences between patients’ phenotypes as concerns disease severity, global cognition and behaviour. Group differences emerged for the FTLD-CDR behavioural domain, with bvFTD patients exhibiting significantly higher behavioural mean scores compared to nfvPPA patients. As expected, all patients that performed the lumbar puncture (*N* = 32) were characterized by a CSF profile not suggestive of AD pathology.

**Table 1:** Sociodemographic and clinical features of FTD patients and healthy controls

	HC (*n* = 48)	bvFTD (*n* = 23)	nfvPPA (*n* = 14)	svPPA (*n* = 17)	rtvFTD (*n* = 13)
Age, years	62.8 (±8.7)	68.1 (±7.7)	67.1 (±10)	63.4 (±9.4)	63.4 (±9.3)
Education, years	12.1 (±3.9)	10.6 (±3.3)	11.9 (±6)	12.9 (±4.3)	10.15 (±3.5)
Sex (M/W)	13/35	14/9^a*^	6/8	9/8	9/4^a*^
Global cognition and behaviour					
MMSE	29.2 (±0.9)	25.2 (±3)^a***^	24.6 (±6.7)^a***^	23.4 (±6.4)^a***^	26.5 (±3.3)^a**^
FAB	–	12.35 (±2.81)	12.36 (±2.84)	13.35 (±3.37)	13.25 (±3.2)
CDR					
Mean, SD	–	0.68 (±0.35)	0.36 (±0.64)	0.53 (±0.35)	0.69 (±0.48)
Frequency (0/0.5/1/2/3)		3/8/8/0/0	8/2/1/1/0	3/8/4/0/0	1/8/3/1/0
CDR-SB	–	4.12 (±2.05)	2.18 (±2.56)	2.6 (±2.35)	3.96 (±3.6)
FTLD-CDR	–	6.19 (±2.96)	4.2 (±3.25)	4.87 (±3.32)	5.95 (±4.63)
FTLD-CDR-behaviour					
Mean, SD	–	1.36 (±0.81)	0.36 (±0.48)^b*^	0.72 (±0.75)	1.31 (±0.59)
Frequency (0/0.5/1/2/3)		1/0/6/3/1	4/1/2/0/0	2/5/0/2/0	0/1/4/3/0
FTLD-CDR-language					
Mean, SD	–	0.64 (±0.74)	0.86 (±0.56)	1.22 (±0.74)	0.56 (±0.32)
Frequency (0/0.5/1/2/3)		4/4/1/2/0	0/4/2/1/0	0/0/7/2/0	1/5/2/0/0
NPI	–	22.28 (±20.83)	12.83 (±19.54)	22.40 (±16.06)	15.75 (±10.93)

Data are presented as mean ± standard deviation. For CDR and FTLD-CDR domains, score frequencies (0/0.5/1/2/3) are additionally reported. ‘a’ indicates a significant difference versus the control group, ‘b’ indicates a significant difference versus the bvFTD group. *P-*values refer to ANOVA models or Fisher’s Exact test (*P* < 0.05). *P*-values were Bonferroni-corrected for multiple comparisons. Statistical significance is indicated by asterisks: *P < 0.05; **P < 0.01; ***P < 0.001. Abbreviations: HC = healthy controls; bvFTD = behavioural variant frontotemporal dementia; nfvPPA = nonfluent variant parimary progressive aphasia; svPPA = semantic variant primary progressive aphasia; rtvFTD = semantic behavioural variant frontotemporal dementia; M = men; W = women; MMSE = Mini-Mental State Examination; FAB = Frontal Assessment Battery; CDR = Clinical Dementia Rating Scale; CDR-SB = CDR sum of boxes; FTLD-CDR = FTLD-Clinical Dementia Rating Scale; SD = standard deviation; NPI = neuropsychiatric inventory.

The neuropsychological features of the sample are reported in the Supplementary Table 1. Patients performed worse than HC in the majority of cognitive domains. SvPPA patients performed worse in semantic fluency compared to the other groups, and in naming compared to bvFTD and nfvPPA cases; nfvPPA patients performed significantly worse in the Digit span backward compared to rtvFTD patients; bvFTD patients committed a higher number of perseverative errors as compared with rtvFTD cases.

**Figure 2:**
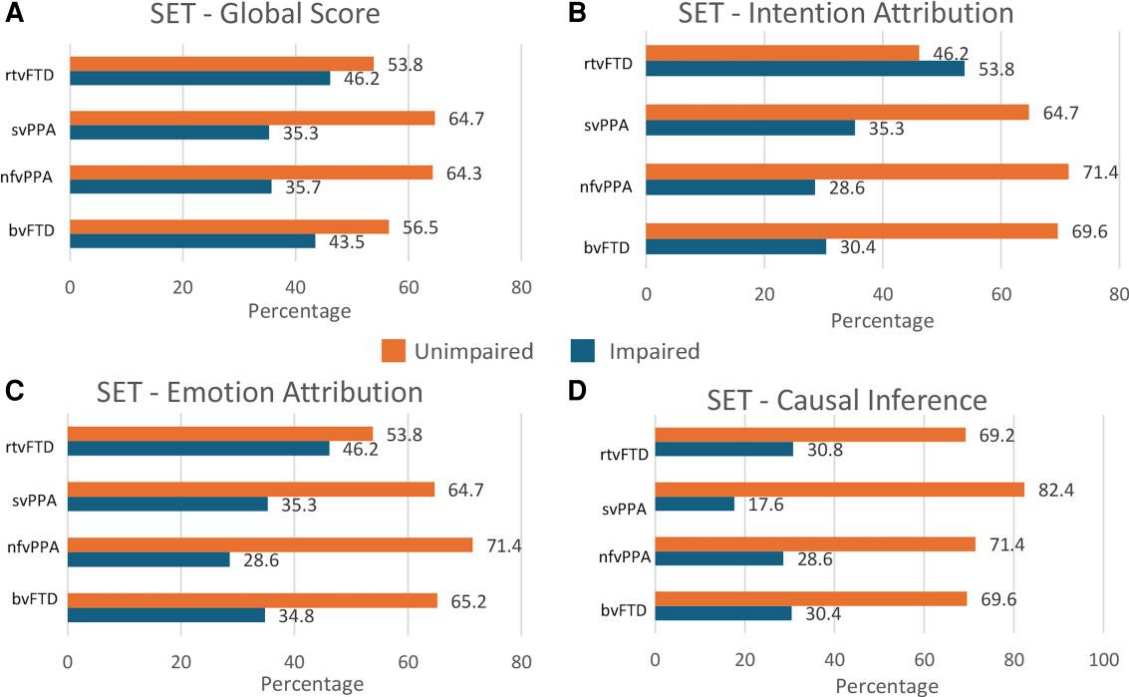
**Percentages of SET pathological scores across patient groups.** Bar plots show the percentages of patients classified as impaired (blue) and unimpaired (orange) on the SET across different patient groups. Panel **A** depicts the SET global score, while panels **B**, **C** and **D** report performance on the SET subtests assessing Intention Attribution, Emotion Attribution and Causal Inference, respectively. Percentages are reported separately for each diagnostic group (bvFTD, nfvPPA, svPPA, rtvFTD; *N* = 23, 14, 17, 13, respectively; experimental unit = patient). Each bar represents the percentage of patients classified as impaired or unimpaired in each patient group. Comparisons of the distribution of pathological scores across groups were performed using Fisher’s exact test (global score: *P* = 0.907; IA: *P* = 0.512; EA: *P* = 0.843; CI: *P* = 0.837). Abbreviations: SET = Story-Based Empathy Task; bvFTD = behavioural variant frontotemporal dementia; nfvPPA = nonfluent variant primary progressive aphasia; svPPA = semantic variant primary progressive aphasia; rtvFTD = right temporal variant frontotemporal dementia.

Global SET mean scores, as well as IA, CI and EA subtest scores, were not different among FTD groups (Supplementary Table 2, Fig. 2). The frequency of patients with SET pathological scores was also similar (Fig. 2).

### Seed-based network definition in young healthy subjects

See Supplementary Fig. 1 for visual representation of each rs-fMRI derived network [affective Tom, cognitive ToM, language (lIFG, lATL), rATL, SN].

Focusing on the specific ToM network reconstruction, seed-based rs-fMRI FC analysis revealed two distinct but interdependent ToM systems. More specifically, we observed that the affective ToM network, centred on the lmPFC, included anterior and ventral regions such as the left supplementary motor area, left temporal pole and, bilaterally, precentral gyrus and insula. On the other hand, the cognitive ToM system built on rSMG encompassed a more posterior and dorsolateral network, which included the right precuneus, left inferior parietal gyrus and middle occipital gyrus, bilateral angular gyrus and cerebellum crus I and II. The other networks reflect a pattern of FC overlapped with those already described in previous literature.^38^ Additionally, as expected, the network centred on the right ATL showed overlapping topography mirroring left ATL network.

### Network analysis

#### Global network analysis

Global functional metrics for each network are summarized in Table 2. Within the affective ToM network, nfvPPA patients exhibited lower nodal strength and higher path length relative to HC, bvFTD and rtvFTD, along with a reduced clustering coefficient compared to HC. In the cognitive ToM network, all patient groups showed increased path length compared to HC, with rtvFTD also displaying reduced nodal strength. As for the right ATL network, both svPPA and rtvFTD cases showed diminished nodal strength relative to HC.

**Table 2:** Global network properties of each network in patients and healthy controls

	HC	bvFTD	nfvPPA	svPPA	rtvFTD
*Affective ToM network*					
Nodal strength	5.40 (±1.82)^b*^	4.88 (±1.59)^b*^	3.36 (±1.14)	5.41 (±2.79)	5.85 (±1.45)^b**^
Path length	5.14 (±1.44)^b**^	5.65 (±1.01)	6.97 (±1.27)	5.64 (±1.67)	5.18 (±1.01)^b*^
Local efficiency	0.24 (±0.07)	0.22 (±0.06)	0.18 (0.05)	0.24 (±0.1)	0.26 (±0.05)
Clustering coefficient	0.11 (±0.03)^b**^	0.09 (±0.02)	0.07 (±0.02)	0.11 (±0.04)	0.11 (±0.03)
*Cognitive ToM network*					
Nodal strength	7.20 (±2.42)	5.40 (±1.86)	5.42 (±1.94)	6.17 (±3.29)	4.47 (±1.48)^a**^
Path length	4.36 (±0.87)	5.77 (±1.24)^a**^	5.43 (±0.99)^a*^	5.70 (3.62)^a*^	6.42 (±3.13)^a**^
Local efficiency	0.35 (±0.09)	0.29 (±0.08)	0.29 (±0.07)	0.31 (±0.13)	0.27 (±0.05)
Clustering coefficient	0.23 (±0.07)	0.19 (±0.06)	0.18 (±0.06)	0.21 (±0.1)	0.19 (±0.05)
*Left ATL network*					
Nodal strength	3.76 (±0.01)	3.73 (±1.26)	3.52 (±1.67)	3.31 (±1.45)	3.69 (±1.10)
Path length	14.5 (±10.2)	20.9 (±32.6)	12.7 (±8.34)	14.4 (±9)	15.7 (±8.26)
Local efficiency	0.32 (±0.07)	0.31 (±0.08)	0.30 (±0.09)	0.30 (±0.1)	0.34 (±0.15)
Clustering coefficient	0.23 (±0.06)	0.23 (±0.07)	0.20 (±0.08)	0.21 (±0.08)	0.25 (±0.12)
*Right ATL network*					
Nodal strength	3.94 (±1.24)	3.03 (±1.1)	3.47 (±1.1)	2.86 (±0.9)^a*^	2.57 (±1.25)^a*^
Path length	7.10 (±2.67)	7.89 (±3.37)	8.39 (±3.05)	8.57 (±4.2)	8.48 (±2.61)
Local efficiency	0.30 (±0.09)	0.25 (±0.07)	0.27 (±0.06)	0.23 (±0.07)	0.23 (±0.07)
Clustering coefficient	0.20 (±0.07)	0.17 (±0.06)	0.17 (±0.05)	0.15 (±0.05)	0.15 (±0.05)
*Left IFG network*					
Nodal strength	5.24 (±1.26)	3.62 (±1.42)^a**^	3.49 (±0.99)^a**^	4.88 (±2.21)	4.96 (±1.63)
Path length	4.97 (±0.92)	6.26 (±1.15)^a**^	6.74 (±1.16)^a**^	5.35 (±1.25)	5.43 (±1.04)
Local efficiency	0.34 (±0.06)	0.26 (±0.06)^a**^	0.24 (±0.06)^a**^	0.33 (±0.12)	0.33 (±0.08)
Clustering coefficient	0.25 (±0.06)	0.19 (±0.05)^a**^	0.17 (±0.06)^a**^	0.25 (±0.11)	0.24 (±0.07)
*SN network*					
Nodal strength	5.55 (±1.55)	3.83 (±1.17)^a**^	4.35 (±1)	5.72 (±2.36)	4.79 (±1.08)
Path length	4.71 (±0.93)	5.99 (±1.15)^a**^	5.97 (±1.19)^a**^	4.75 (±1.08)	5 (±0.83)
Local efficiency	0.32 (±0.08)	0.24 (±0.06)^a*^	0.25 (±0.04)	0.33 (±0.13)	0.30 (±0.06)
Clustering coefficient	0.19 (±0.06)	0.14 (±0.04)^a*^	0.14 (±0.03)	0.20 (±0.10)	0.18 (±0.05)

Data are presented as mean ± standard deviation. ‘a’ indicates a significant difference versus the control group, ‘b’ indicates a significant difference versus the nfvPPA group. *P-*values refer to ANCOVA models on rank-transformed data. *P*-values were Bonferroni-corrected for multiple comparisons; to control for multiple testing across global properties, a family-wise correction was applied considering the number of network indexes assessed. Statistical significance is indicated by asterisks: *P < 0.05; **P < 0.01; ***P < 0.001. Abbreviations: HC = Healthy Controls; bvFTD = behavioural variant frontotemporal dementia; nfvPPA = nonfluent variant primary progressive aphasia; svPPA = semantic variant primary progressive aphasia; rtvFTD = right temporal variant frontotemporal dementia; ATL = anterior temporal lobe; IFG = inferior frontal gyrus; SN = salience network.

In the left IFG and SN networks, bvFTD patients showed reduced nodal strength, local efficiency and clustering coefficient, as well as increased path length; nfvPPA cases displayed increased path length in both networks, with additional reductions in nodal strength, local efficiency and clustering in the left IFG. No significant differences among groups were observed in the left ATL network.

#### Connectivity analysis

**Figure 3:**
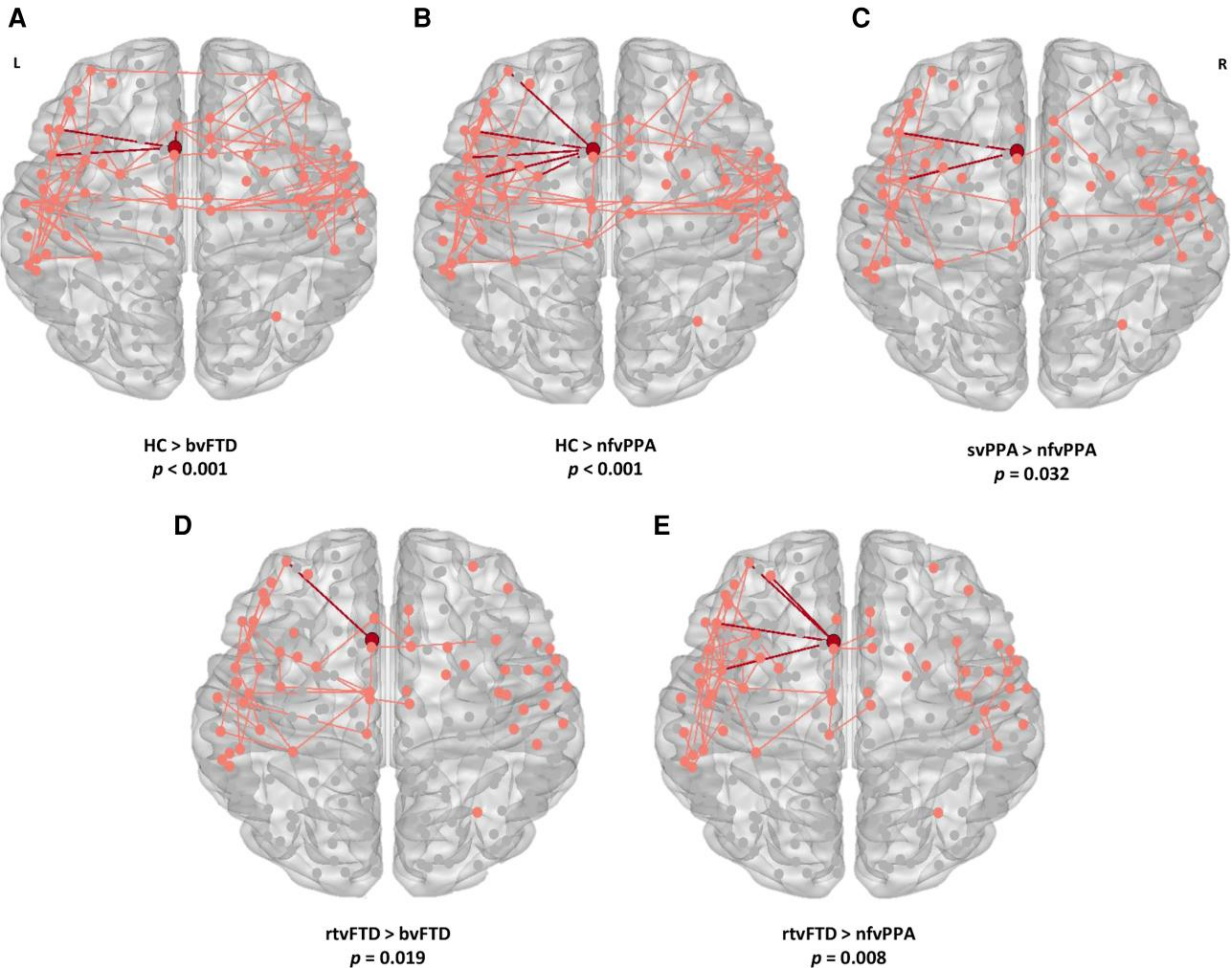
**Between-group differences in network-based regional connectivity and direct connections from seed within the affective ToM network.** Connections showing reduced functional connectivity (FC) across comparisons: bvFTD (*N* = 23) versus HC (*N* = 48; panel **A**), nfvPPA (*N* = 14) versus HC (panel **B**), nfvPPA versus svPPA (*N* = 17; panel **C**), bvFTD versus rtvFTD (*N* = 13; panel **D**) and nfvPPA versus rtvFTD (panel **E**). Edges were included in a network component if they showed a significant difference between groups (*P* < 0.05, ANCOVA). Component-level significance was determined via 5000 permutations, controlling for family-wise error (FWE). The reported component intensity corresponds to the sum of t-statistics across edges within the component. Each dot represents a node within the functional network. Light-coloured lines indicate all connections significantly altered within the identified network and for the given contrast, whereas darker lines highlight direct connections departing from the seed region (seed region for the affective ToM Network = left medial prefrontal cortex). The reported *P*-values refer to significant effects at the network level and are permutation-corrected and adjusted for age, sex and education. Please note that no statistical inference is made at the level of direct seed connections. Abbreviations: HC = healthy controls; bvFTD = behavioural variant frontotemporal dementia; nfvPPA = nonfluent variant primary progressive aphasia; svPPA = semantic variant primary progressive aphasia; rtvFTD = right temporal variant frontotemporal dementia.

**Figure 4:**
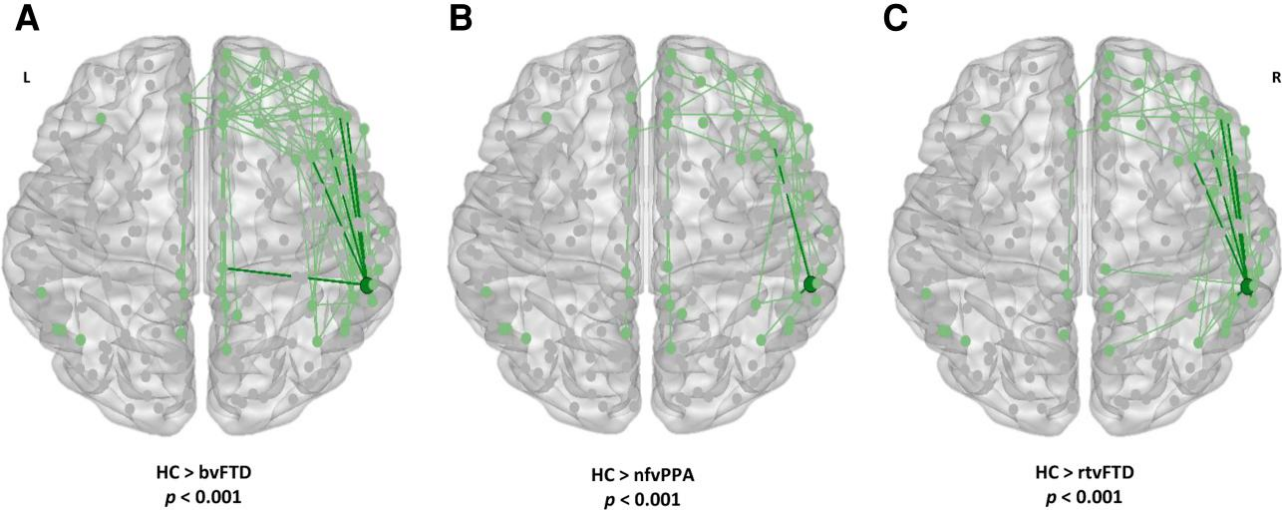
**Between-group differences in network-based regional connectivity and direct connections from seed within the cognitive ToM network.** Connections show reduced functional connectivity (FC) across comparisons: bvFTD (*N* = 23) versus HC (*N* = 48; panel **A**), nfvPPA (*N* = 14) versus HC (panel **B**), rtvFTD (*N* = 13) versus HC (panel **C**). Edges were included in a network component if they showed a significant difference between groups (*P* < 0.05, ANCOVA). Component-level significance was determined via 5000 permutations, controlling for family-wise error (FWE). Each dot represents a node within the functional network. Light-coloured lines indicate all connections significantly altered within the identified network and for the given contrast, whereas darker lines highlight direct connections departing from the seed region (seed region for the cognitive ToM network = right supramarginal gyrus). The reported *P*-values refer to significant effects at the network level and are permutation-corrected and adjusted for age, sex and education. Please note that no statistical inference is made at the level of direct seed connections. Abbreviations: HC = healthy controls; bvFTD = behavioural variant frontotemporal dementia; nfvPPA = nonfluent variant primary progressive aphasia; svPPA = semantic variant primary progressive aphasia; rtvFTD = right temporal variant frontotemporal dementia.

Concerning NBS, patterns of decreased seed-centred connectivity in each group of FTD patients compared to HC and for affective and cognitive ToM networks are reported in Figs 3 and 4. Patterns of decreased FC for the other large-scale networks are reported from Supplementary Figs 3–6.

Between-group whole network differences are detailed in the Supplementary material. For each network, the direct connections from the seed region to the rest of the network were plotted in HC (Supplementary Fig. 2).

The FC alterations within the direct connections departing from each seed of each network are reported in the following sections.

##### Affective ToM network

When considering the direct connections from the seed region, as compared with HC, bvFTD patients displayed reduced connectivity with the pars opercularis and triangularis of the left IFG and with the left ACC. As compared to HC, nfvPPA patients exhibited the same pattern of reduced FC, with the additional involvement of the connectivity between the seed and the left precentral and middle frontal gyri, left middle cingulate gyrus and left putamen. No significant differences emerged when comparing HC and both svPPA and rtvFTD patients.

Significant differences also appeared between patient groups. As compared with svPPA and rtvFTD cases, nfvPPA patients displayed a pattern of reduced connectivity mainly involving left-lateralized regions such as the precentral, middle and IFG. Reduced connectivity from the seed to the left middle frontal gyrus was observed in bvFTD cases as compared to rtvFTD patients (Fig. 3).

##### Cognitive ToM network

As compared with HC, direct connections from seed appeared altered in bvFTD, nfvPPA and rtvFTD cases. Specifically, bvFTD patients exhibited reduced connectivity with the right inferior and middle frontal gyrus, right insula and middle cingulate cortex. rtvFTD cases showed reduced connectivity to the right inferior and middle frontal gyrus, right insula, right middle temporal gyrus and right inferior parietal gyrus. Conversely, nfvPPA cases only displayed reduced connectivity with the right middle frontal gyrus and right inferior parietal gyrus (Fig. 4). No significant differences were observed when comparing svPPA patients to the HC group, nor among patient groups.

##### Left ATL network

Compared to HC, svPPA cases displayed reduced connections from seed to left middle and inferior temporal gyri (Supplementary Fig. 3). No significant differences were observed when comparing the other patient groups to the HC group, nor among patient groups.

##### Right ATL network

When focusing on direct connections from the seed region, both svPPA and rtvFTD patients showed reduced connectivity with the right middle temporal gyrus. In bvFTD patients, this pattern was similarly observed, with the additional involvement of the connectivity between the seed and the right inferior temporal gyrus (Supplementary Fig. 4).

##### Left IFG network

Exploring the direct connectivity stemming from the seed revealed altered FC patterns in bvFTD, nfvPPA and rtvFTD patients as compared to HC. In bvFTD, left IFG exhibited altered FC with the left precentral gyrus, the left pars opercularis and triangularis of the IFG and the left superior medial frontal gyrus. A similarly affected pattern characterized nfvPPA cases, with further implication of the left insula and left putamen. In contrast, rtvFTD patients only displayed altered FC from seed to the left pars orbitalis of the IFG. NfvPPA patients exhibited disrupted FC when compared to both svPPA and rtvFTD cases. Specifically, when compared to the svPPA group, nfvPPA patients displayed altered connectivity from the seed to the left insula. A similar pattern was observed when comparing nfvPPA to rtvFTD patients, with the additional involvement of the left precentral gyrus.

Abnormal FC stemming from seed to the left insula and left precentral gyrus also characterized bvFTD patients when compared to the rtvFTD group (Supplementary Fig. 5).

##### Salience network

Compared to the HC group, bvFTD patients exhibited reduced connectivity from the seed region, affecting both intra- and interhemispheric pathways. Specifically, decreased connectivity was observed with the right middle frontal gyrus, supplementary motor area, insula, middle cingulate cortex and the left anterior cingulate cortex. Similarly, nfvPPA patients showed reduced connectivity from the seed to the right insula, right middle cingulate cortex, left anterior cingulate cortex and right thalamus. In contrast, rtvFTD patients exhibited a more restricted pattern of reduced connectivity, limited to the right insula and left anterior cingulate cortex.

Finally, compared to rtvFTD patients, both bvFTD and nfvPPA cases displayed weakened connectivity from the seed region to the right middle cingulate cortex (Supplementary Fig. 6).

### Correlation between functional connectivity and neuropsychological data

#### Correlations with global network properties

In all patients, a negative relationship was observed between the total score of the FBI and clustering coefficient of the SN network (*r* = −0.36; *P* = 0.01). Moreover, the SET global score correlated with nodal strength (*r* = 0.35; *P* = 0.003), local efficiency (*r* = 0.32; *P* = 0.01) and clustering coefficient (*r* = 0.32; *P* = 0.01) of the cognitive ToM network.

In the bvFTD group, the Token test was positively associated with nodal strength (*r* = 0.64; *P* = 0.001) and local efficiency (*r* = 0.54; *P* = 0.01) within the SN network, while it was negatively correlated with path length (*r* = −0.63; *P* = 0.002) within the same network. SET global score was related to higher nodal strength (*r* = 0.53; *P* = 0.01) within the cognitive ToM network.

NfvPPA patients displayed a positive relationship between the Token test and path length (*r* = 0.83; *P* = 0.002) of the affective ToM network.

No significant relationships emerged when considering the svPPA and the rtvFTD group.

#### Correlations with the mean functional connectivity from the seed

No significant correlations were observed between mean FC and cognitive test scores in the overall sample. However, when analysing the data separately for each patient group, we observed significant correlations in the bvFTD group. Specifically, a positive correlation was found between the Token test score and the mean connectivity of the SN network (*r* = 0.57, *P* = 0.01), as well as between the SET global score and the mean connectivity of the cognitive ToM network (*r* = 0.55, *P* = 0.01). No significant relationships emerged for the other groups.

## Discussion

The present study investigated cognitive and affective ToM in a broad population of FTD patients, including bvFTD, rtvFTD and PPA cases, and determined whether these abilities are equally impaired across the different clinical variants. Although ToM deficits in bvFTD patients have been extensively studied, limited research has explored these abilities in PPA cases, and, to our knowledge, no study has directly compared ToM performance across these four FTD clinical subtypes. Additionally, although neuroimaging studies have sought to identify brain regions underlying ToM, no consensus has been reached regarding the neural networks involved or their specific roles in cognitive versus affective ToM processes. For these reasons, we aimed at reconstructing brain networks mediating cognitive and affective ToM, as well as at delineating the patterns of global and regional FC deterioration, and at evaluating differences and commonalities across clinical syndromes. Four main findings emerged: first, cognitive and affective ToM abilities appeared equally hampered in all patient groups; second, seed-based rs-FC analysis revealed the existence of two distinct, but interdependent ToM subsystems; third, each FTD variant showed FC abnormalities in the specific networks underlying the core symptoms characterizing the disorder; fourth, bvFTD and nfvPPA cases demonstrated additional significant alterations in the functional networks mediating cognitive and affective ToM.

We observed that both cognitive and affective ToM abilities, respectively, assessed by the IA and EA subscales of the SET, were equally impaired across FTLD variants. Patients exhibited an overall deterioration in both cognitive and affective components irrespective of the clinical phenotype, and differences in pathological scores across patient groups (bvFTD, nfvPPA, svPPA, rtvFTD) emerged as non-significant. Compared to previous works assessing ToM competencies in PPA, this study is innovative for several reasons. First, our findings suggest that the neuropsychological features characterizing PPA are not constrained to language impairments^6^ and challenge the prevailing notion that ToM deficits are specific to bvFTD. As all patient groups had comparable disease severity and the majority of patients were classified in the very mild stage (CDR = 0/0.5), these results point to a shared vulnerability of ToM networks across the FTLD spectrum,^44,45^ supporting the hypothesis that ToM difficulties may represent an early hallmark of the disease. Second, it is worth noting that patients performed poorly on the SET, which is designed to reduce the influence of language on performance by making use of visual but non-verbal stimuli, suggesting at least a partial independence of ToM deficits from language impairment. This aligns with prior research demonstrating early and significant socio-cognitive impairments across the FTD spectrum, regardless of the primary clinical presentation.^6,46,47^

In this study, we provide critical evidence linking ToM deficits to disruptions in FC within ToM-related networks. Across the FTD spectrum, distinct patterns of network alterations were observed, reflecting the clinical and cognitive heterogeneity of these disorders. By employing a seed-based rs-FC analysis, we were able to identify two distinct yet interdependent ToM systems, each characterized by unique spatial and functional properties.

The affective ToM network showed predominant involvement of ventral brain regions, including the left supplementary motor area, left temporal pole and, bilaterally, the precentral gyrus and insula. This ventral system aligns with prior studies emphasizing its role in processing emotional aspects of ToM, such as empathy and emotion recognition.^44,48,49^ In contrast, the cognitive ToM network exhibited a more posterior and dorsolateral distribution, involving the right precuneus, left inferior parietal gyrus, middle occipital gyrus, bilateral angular gyrus and cerebellum (crus I and II). These regions are associated with mentalizing and perspective-taking, reflecting their involvement in inferring others’ beliefs and intentions.^44,48^

When focusing specifically on ToM networks, we observed the most extensive disruptions in bvFTD and nfvPPA, affecting both cognitive and affective components, and involving key hubs such as the mPFC and insula. These widespread alterations are consistent with the severe socio-cognitive impairments that characterize these syndromes and suggest that ToM network dysfunctions may represent a hallmark of these subtypes.^44,47^ In bvFTD, the pattern of widespread atrophy across frontal, temporal and subcortical regions, which are involved in social, cognitive and executive functions, results in profound socio-emotional deficits and behavioural disinhibition.^3,50^ Similarly, neurodegeneration in nfvPPA affects networks critical for speech and executive control while sparing more posterior regions involved in cognitive ToM, such as the angular gyrus and precuneus. By contrast, affective ToM appears more vulnerable, likely due to the degeneration of the left IFG and its connections with mPFC and insula, regions involved in emotional processing and social cognition.^6,44,50,51^ This pattern aligns with growing evidence of functional specialization within the lIFG, where dorsal portions contribute to mirroring processes relevant for affective ToM, whereas ventral regions appear to support cognitive ToM by enabling self-perspective inhibition.^10,48,52,53^

In contrast to bvFTD and nfvPPA, svPPA and rtvFTD exhibited relatively preserved connectivity within both cognitive and affective ToM networks. This preservation is consistent with their more focal neurodegenerative profile, primarily centred on the ATL, as opposed to the broad frontotemporal and subcortical involvement observed in bvFTD cases.^5,44,54^

The diverse FC patterns observed across FTD variants may reflect differences in their most frequently, though not exclusively, reported neuropathological substrates. svPPA and rtvFTD are commonly associated with type C TDP-43 pathology, which typically shows focal temporal involvement.^42,55^ In contrast, nfvPPA is more often linked to 4-repeat tau (4R tau) pathology.^56^ As concerns bvFTD, neuropathology is more heterogeneous, with both tau and TDP-43 frequently reported.^57^ The variability observed in FC alterations across variants may partly reflect these distinct pathological profiles. Nevertheless, neuropathological differences alone do not fully account for the heterogeneity observed at the network level. Recent evidence from rtvFTD shows that rATL degeneration is reflected not only by local atrophy but also by large-scale reorganization of functional networks^58^ suggesting that network-level adaptations may play a critical role in shaping socio-emotional and cognitive symptoms in FTD. Clarifying the extent to which such secondary adaptations shape FC patterns across FTD subtypes remains an important direction for future research. The observed correlations between FC metrics and SET performance further highlight the critical role of ToM networks in mediating socio-cognitive deficits. Across all clinical subtypes, disruptions in the cognitive ToM network were associated with global SET scores, emphasizing the relevance of these networks in ToM functioning. Notably, in bvFTD, connectivity alterations within both cognitive and affective ToM components correlated with socio-cognitive measures. In nfvPPA, affective ToM connectivity was associated with linguistic measures, such as the Token test. Conversely, in rtvFTD, connectivity within the right ATL network showed a significant relationship with SET-EA scores. These findings suggest that the socio-cognitive impairments observed in these patients may be modulated, at least in part, by their primary functional and clinical deficits, reflecting a close interaction between social cognition and other cognitive domains. In nfvPPA, this interaction may involve language comprehension processes that are supported by working memory resources, which are engaged by tasks such as the Token test and are critical for maintaining and integrating information over time.^59^ More broadly, this pattern highlights the complex neural and cognitive interdependencies underlying these syndromes. In this context, the relative preservation of affective and cognitive ToM networks in rtvFTD and svPPA cases may indicate that impaired SET performance in these variants could be partially influenced by non-social cognitive deficits. Our findings suggest that, at least in some FTD variants, SET scores should be interpreted with caution and within a broader neurocognitive framework.

This study is not without limitations. First of all, the relatively small sample size for each FTD subtype reduces statistical power and limits generalizability, particularly for correlation analyses. Second, we cannot entirely exclude the influence of language deficits on the performance of svPPA patients. Indeed, these patients do suffer from a severe failure of semantic knowledge, which may affect their ability to identify the object itself. Third, the absence of patients with the logopenic variant of PPA (lvPPA), typically linked to Alzheimer's pathology, limits our ability to directly compare FTD- and AD-related network alterations. Future research should address these limitations by incorporating larger cohorts and multimodal imaging approaches, including structural connectivity analyses, to provide a more comprehensive understanding of network disruptions in FTD.

In conclusion, this study underscores the centrality of ToM impairments across the FTD spectrum and their neural underpinnings in distinct network disruptions. By elucidating the relationship between FC and socio-cognitive deficits, our findings advance the understanding of FTD pathophysiology and pave the way for targeted diagnostic and therapeutic strategies.

## Supplementary Material

fcag216_Supplementary_Data

## Data Availability

The anonymized dataset used and analysed during the current study will be made available by the corresponding author upon request to qualified researchers (i.e. affiliated to a university or research institution/hospital). Seed-based analyses were conducted using standard tools implemented in FSL. For the connectomics analysis, the codes that support the findings of this study are openly available in GitHub public repository at https://github.com/SilviaBasaia/Theory-of-mind-in-frontotemporal-dementia.
