# Supplementary material for: Affective and cognitive theory of mind and associated brain functional alterations in frontotemporal dementia
Source: Brain Commun. 2026 Jun 6;8(3):fcag216. doi: 10.1093/braincomms/fcag216 (PMC13270490; doi:10.1093/braincomms/fcag216)
Supplement: fcag216_Supplementary_Data [file fcag216_supplementary_data.pdf]

Supplementary Material

Results

Network-Based connectivity

Affective ToM network

Within the affective ToM network and as compared with HC, bvFTD patients displayed a bilateral pattern of reduced connectivity mainly involving intra- and interhemispheric connections between the sensorimotor, frontal, temporal, insular and basal ganglia (putamen) nodes. The same pattern of reduced functional connectivity was observed in nfvPPA cases as compared to HC, with the additional involvement of left parietal and supramarginal nodes. By comparing rtvFTD patients and HC, a prominent deterioration of right frontal and insular connections emerged. Differences between the svPPA and control group were non-significant.

Significant differences also appeared when comparing patient groups. NfvPPA cases displayed a pattern of reduced connectivity mainly involving connections between left frontal, sensorimotor and temporal nodes as compared with svPPA cases, whereas they demonstrated a prevalent reduced connectivity between left frontal, sensorimotor and parietal regions as compared with rtvFTD patients. Moreover, as compared with the rtvFTD group, bvFTD patients displayed disrupted connectivity affecting fronto-insular, fronto-opercular, and premotor–sensorimotor pathways, especially in the left hemisphere.

Cognitive ToM network

The comparison of bvFTD, nfvPPA, and rtvFTD patients, separately, with HC revealed widespread patterns of reduced functional connectivity mainly involving right long-range intrahemispheric connections between frontal, temporal, parietal, and insular regions. In contrast, the svPPA group did not exhibit any significant differences compared to HC.

Compared to svPPA and rtvFTD cases, bvFTD patients also demonstrated reduced functional connectivity involving right intrahemispheric connections between frontal, temporal, and parietal nodes, and between right anterior cingulum and precuneus.

Left ATL network

As compared with HC, bvFTD, svPPA and rtvFTD groups demonstrated decreased functional connectivity involving left short-range intrahemispheric connections between mesial (including

hippocampus and amygdala), middle and superior temporal regions, as well as lingual, and angular nodes.

Right ATL network

Compared to HC, the groups showing significantly altered functional connectivity within the right ATL network were bvFTD, svPPA and rtvFTD. In particular, bvFTD patients showed decreased connectivity in bilateral superior frontal as well as the left inferior and superior temporal connections. In the rtvFTD and svPPA group, connectivity was predominantly altered in bilateral middle temporal connections in the first group, and in right frontal, left medial and inferior temporal regions, as well as left amygdala and parahippocampal connections in the second group.

Left IFG network

With respect to HC, bvFTD patients showed a predominantly left-lateralized loss of functional connectivity, mostly localized in the inferior frontal, precentral, superior temporal, anterior and posterior cingulate regions, as well as putamen. Additionally, some interhemispheric connections between superior frontal areas and the insula were also found to be affected.
A similar pattern of altered connectivity was observed also in rtvFTD and nfvPPA cases as compared with HC. Moreover, rtvFTD patients displayed an overall more preserved functional connectivity as compared to bvFTD and nfvPPA groups, in particular in left-lateralized sensorimotor, temporal, and insular regions. In contrast, nfvPPA cases exhibited more pronounced disruptions than svPPA, involving a broader left-lateralized network that encompassed sensorimotor, frontal, temporal, limbic, and subcortical areas including the thalamus, putamen, and insula. SvPPA emerged as the less affected group.

SN

A widespread pattern of decreased functional connectivity in bilateral sensorimotor, cingulate, insular and parietal connections, extending to thalamic and putamen connections characterized bvFTD patients when compared to the HC. The same pattern was observed when comparing HC with nfvPPA and rtvFTD cases, who exhibited less extended connectivity alterations in bilateral superior and middle frontal regions, anterior cingulate, thalamus, and basal ganglia with respect to the other two variants.
Finally, compared to rtvFTD, both bvFTD and nfvPPA patients showed disrupted functional connectivity involving bilateral frontal and subcortical regions, including the thalamus, caudate, putamen, and pallidum. Both groups exhibited altered connections in the anterior and middle

cingulate cortices and supplementary motor areas. However, bvFTD disruptions were more focused on fronto-subcortical and fronto-cingulate pathways, while nfvPPA showed more widespread intra- and inter-hemispheric frontal disconnections.

Supplementary Table 1. Neuropsychological profile of patients

	HC	bvFTD	nfvPPA	svPPA	rtvFTD
Verbal Memory					
**RAVLT (immediate recall) (0-75)**	48.93 (± 6.17)	26.96 (± 7.49)[a**]	30 (± 13.91)[a**]	24.80 (± 10.11)[a**]	27.33 (± 10.08)[a**]
**RAVLT (delayed recall) (0-15)**	10.74 (± 2.19)	3.22 (± 3.07)[a**]	6.18 (± 3.99)[a**]	3.20 (± 3.26)[a**]	4 (± 3.19)[a**]
**RAVLT (recognition) (0-15)**	14.35 (± 1.08)	11.55 (± 4.07)	12.86 (± 2.97)	11.18 (± 2.14)[a**]	10.38 (± 3.42)[a**]
**Digit span, forward (0-9)**	5.89 (± 1.02)	5.13 (± 1.25)	4.14 (± 0.66)[a**]	5.31 (± 1.01)	5.38 (± 1.04)
Spatial Memory					
**Benson figure recall (0-16)**	11.76 (± 2.50)	5.10 (± 3.82)[a**]	7.65 (± 3.98)	8.19 (± 4.56)	7.45 (± 2.5)
**Spatial span (0-10)**	5.38 (± 1.11)	4.09 (± 1.34)[a**]	4.38 (± 1.12)	4.59 (± 1.12)	4.46 (± 1.51)

Abstract Reasoning, Executive Functions and Attention					
**RCPM (0-36)**	32.15 (± 3.46)	20.86 (± 7.5)[a**]	23.92 (± 7.86)[a**]	25.13 (± 8.29)[a**]	24.75 (± 7.57)[a**]
**Attentive Matrices (0-60)**	51.82 (± 6.66)	42.48 (± 10.50)[a**]	38.93 (± 11.82)[a**]	42 (± 11.90)[a*]	48.33 (± 6.79)
**Phonemic fluency**	37.70 (± 8.19)	19.39 (± 11.32)[a**]	10.7 (± 7.37)[a**]	20.25 (± 9.99)[a**]	17.92 (± 7.61)[a**]
**MCST – categories (0-6)**	4.26 (± 1.32)	2.44 (± 1.54)[a*]	3.50 (± 2.14)	4.57 (± 1.50)	4.11 (± 2.47)
**MCST – perseverative errors (0-47)**	3.85 (± 3.75)	15 (± 9.42)[ae**]	11.67 (± 11.76)	4.21 (± 3.42)	3.56 (± 5.6)
**TMT-A**	34.03 (± 11.08)	66.82 (± 25.15)[a**]	60.95 (± 19.94)[a**]	61.82 (± 32.08)[a**]	54.68 (± 19.58)[a**]
**TMT-B**	97.45 (± 45.12)	259.72 (± 148.20)[a**]	167.89 (± 56.80)[a**]	158.25 (± 61.50)[a**]	166.91 (± 49.5)[a**]
**TMT-BA**	63.42 (± 37.83)	190.74 (± 148.57)[a**]	109.22 (± 49.76)	102.81 (± 55.85)	112.23 (± 37.97)
**Digit span, backward (0-8)**	4.93 (± 1.23)	3.55 (± 0.8)[a**]	2.50 (± 1.29)[ae**]	3.27 (± 1.28)[a**]	4 (± 1.04)
**Clock drawing test (0-8)**	-	5.52 (± 3.60)	6.46 (± 3.43)	5.20 (± 3.80)	7 (± 2.9)

Language					
**Token Test (0-36)**	34.37 (± 1.40)	27.80 (± 6.12)[a**]	26.95 (± 4.77)[a**]	26.32 (± 9.91)[a**]	30.17 (± 5.27)[a*]
**Semantic fluency**	47.5 (± 9.61)	20.96 (± 6.50)[a**d*]	23.69 (± 10.44)[ad**]	12.19 (± 6.27)[a**]	23.54 (± 11.36)[ad**]
**CaGi, naming (0-48)**	-	42.75 (± 4.2)[d*]	40.75 (± 12.76)[d**]	21.21 (± 11.83)	33.92 (± 13.87)
Visuospatial Abilities					
**Benson figure copy (0-16)**	15.76 (± 0.78)	14.33 (± 2.33)	13.62 (± 3.43)	14.50 (± 2.07)	15.45 (± 1.21)
**CD (0-12)**	10.24 (± 1.74)	9.23 (± 1.90)	8.33 (± 2.46)	10.19 (± 1.51)	10.08 (± 0.95)
**CDP (0-70)**	67.20 (± 4.01)	62.50 (± 4.60)	63.67 (± 7.40)	65.69 (± 5.53)	66.08 (± 6.07)
Emotion Recognition					
**CATS – Identity discrimination (0-12)**	11.62 (± 0.61)	9.39 (± 2.1)[a**]	9.92 (± 3.60)	10.94 (± 1.53)	11.09 (± 1.14)
**CATS – Affect**	11.29 (± 0.76)	9.61 (± 1.67)[a**]	9.08 (± 3.29)	10.38 (± 1.90)	11.09 (± 1.04)

**discrimination (0-12)**					
**CATS – Affect naming (0-6)**	4.62 (± 1.07)	2.36 (± 1.40)^{a**}	2.83 (± 1.70)^{a**}	3.25 (± 1.95)^{a*}	2.55 (± 1.21)^{a**}
**CATS – Select affect (0-6)**	5.62 (± 0.68)	3.73 (± 1.32)^{a**}	3.83 (± 1.80)^{a**}	4.25 (± 1.24)^{a**}	3.17 (± 0.94)^{a**}
**CATS – Affect matching (0-12)**	9.02 (± 1.89)	6.27 (± 1.80)^{a**}	5 (± 3.22)^{a**}	7.56 (± 1.90)	5.92 (± 1)^{a**}
**CATS – Three faces test (0-24)**	14.09 (± 2.78)	10.32 (± 2.66)^{a**}	7.67 (± 3.96)^{a**}	11.75 (± 3.04)	9.92 (± 1.88)^{a**}
**CATS – Affect Recognition Quotient (0-60)**	80.21 (± 18.14)	32.73 (± 21.74)^{a**}	30.23 (± 26.58)^{a**}	48.78 (± 25.67)^{a**}	29.91 (± 11.57)^{a**}

Values are reported as mean ± standard deviation computed on raw scores. The minimum and maximum scores for each test are indicated in parentheses where a theoretical score range is defined. For tests without a predefined score range, minimum and maximum values are not applicable. Superscript letters (a, b, c) indicate significant pairwise differences between groups. For instance, "a" indicates a significant difference versus the control group, "b" versus the bvFTD group, "c" versus the nfvPPA group, "d" versus the svPPA group, and "e" versus the rtvFTD group. P values refer to ANCOVA models on rank transformed data. P-values were Bonferroni-corrected for multiple comparisons;

*to control for multiple testing across domains, a family-wise correction was applied considering the number of domains assessed. Statistical significance is indicated by asterisks: *p<0.05; **p<0.01; ***p<0.001.*

Abbreviations: HC = Healthy Controls; bvFTD = behavioral variant Frontotemporal Dementia; nfvPPA = nonfluent variant Primary Progressive Aphasia; svPPA = semantic variant Primary Progressive Aphasia; rtvFTD = right temporal variant Frontotemporal Dementia; RAVLT = Rey Auditory Verbal Learning Test; RCPM = Raven's Colored Progressive Matrices; MCST: Modified Card Sorting Test; TMT: Trial Making Test; CaGi = Italian battery for the assessment of semantic memory disorders; CATS = Comprehensive Affect Testing System.

Supplementary Table 2. Story-Based Empathy Task raw scores

	bvFTD	nfvPPA	svPPA	rtvFTD	p-values
**SET-GS**	9.6 (± 3.2)	10.2 (± 4.7)	10.2 (± 5)	9.2 (± 3)	0.973
**SET-IA**	3.4 (± 1.3)	3.4 (± 1.9)	3.3 (± 2)	2.7 (± 1.5)	0.747
**SET-CI**	3.1 (± 1.6)	3.4 (± 1.6)	3.9 (± 1.6)	3.5 (± 1.6)	0.753
**SET-EA**	3.1 (± 1.3)	3.4 (± 1.9)	3 .2 (± 1.8)	3 (± 1.6)	0.943

Values are reported as mean ± standard deviation computed on raw scores. The possible score range for each test is indicated in parentheses. P values refer to ANOVA models. Abbreviations: SET-GS = Story-Based Empathy Task – Global Score; SET-IA = Story-Based Empathy Task – Intention Attribution; SET-CI = Story-Based Empathy Task – Causal Inference; SET-EA = Story-Based Empathy Task – Emotion Attribution.

Supplementary Figure 1. Seed-based functional connectivity network reconstruction

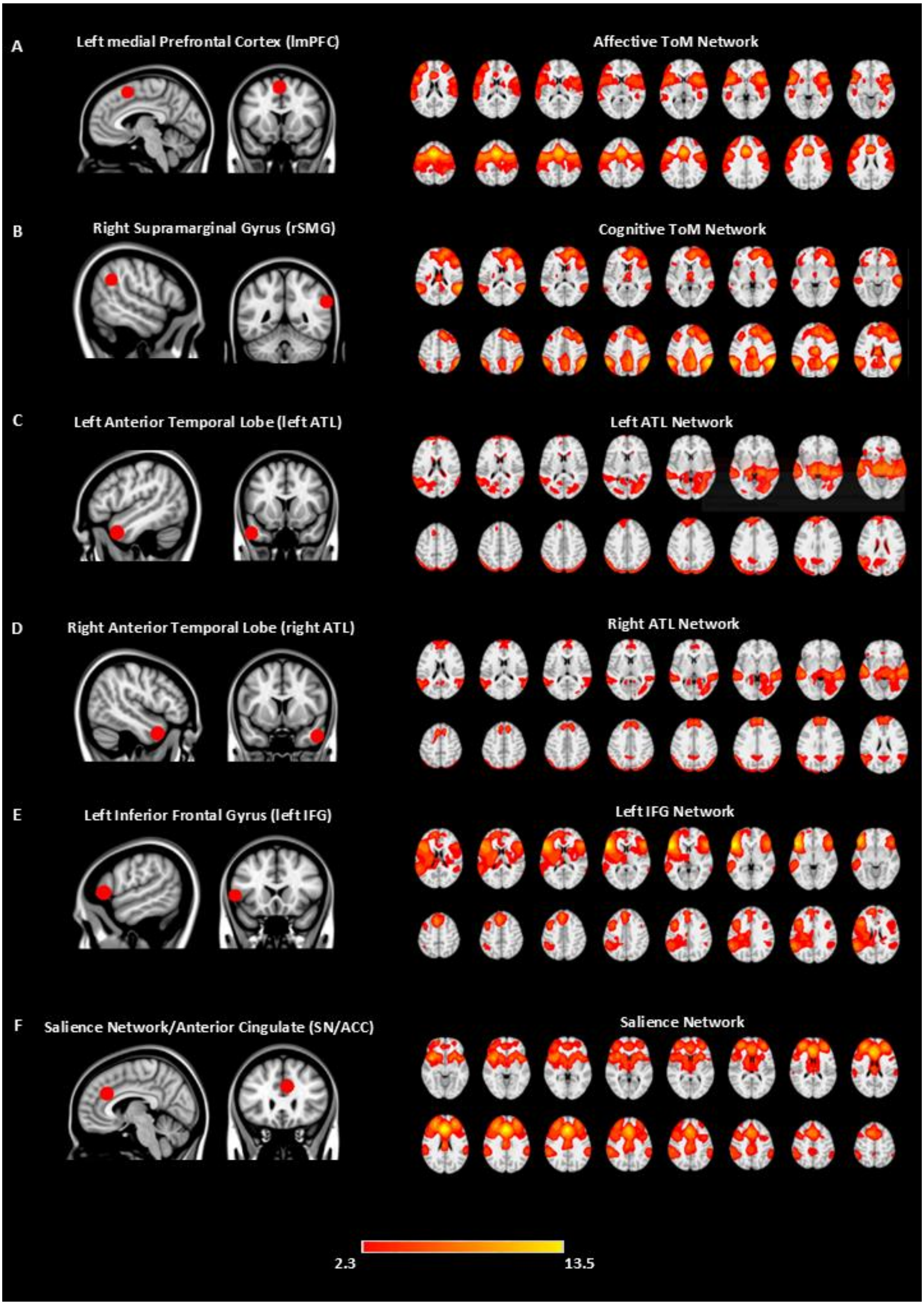

Seed-based functional connectivity maps for six regions of interest are shown. Connectivity was computed by correlating each seed's time series with all brain voxels at the single-subject level. Group-level maps represent the average connectivity across 50 healthy young participants. Z (Gaussianised T/F) statistic images were thresholded using clusters determined by $Z > 2.3$ and a corrected cluster significance threshold of $p = 0.05$. Color bars represent z values. Networks are displayed in MNI space on a standard brain template. N = 50 participants; experimental unit = single participant.

A: Left medial prefrontal cortex (lmPFC) seed and associated affective Theory of Mind (ToM) network; B: Right supramarginal gyrus (rSMG) seed and associated cognitive ToM network; C: Left anterior temporal lobe (left ATL) seed and associated left ATL network; D: Right anterior temporal lobe (right ATL) seed and associated right ATL network; E: Left inferior frontal gyrus (left IFG) seed and associated left IFG network; F: Anterior cingulate cortex (ACC) seed and associated salience network (SN/ACC).

Supplementary Figure 2. Direct connections departing from each seed region to the rest of the network, shown for each of the six large-scale networks in healthy controls.

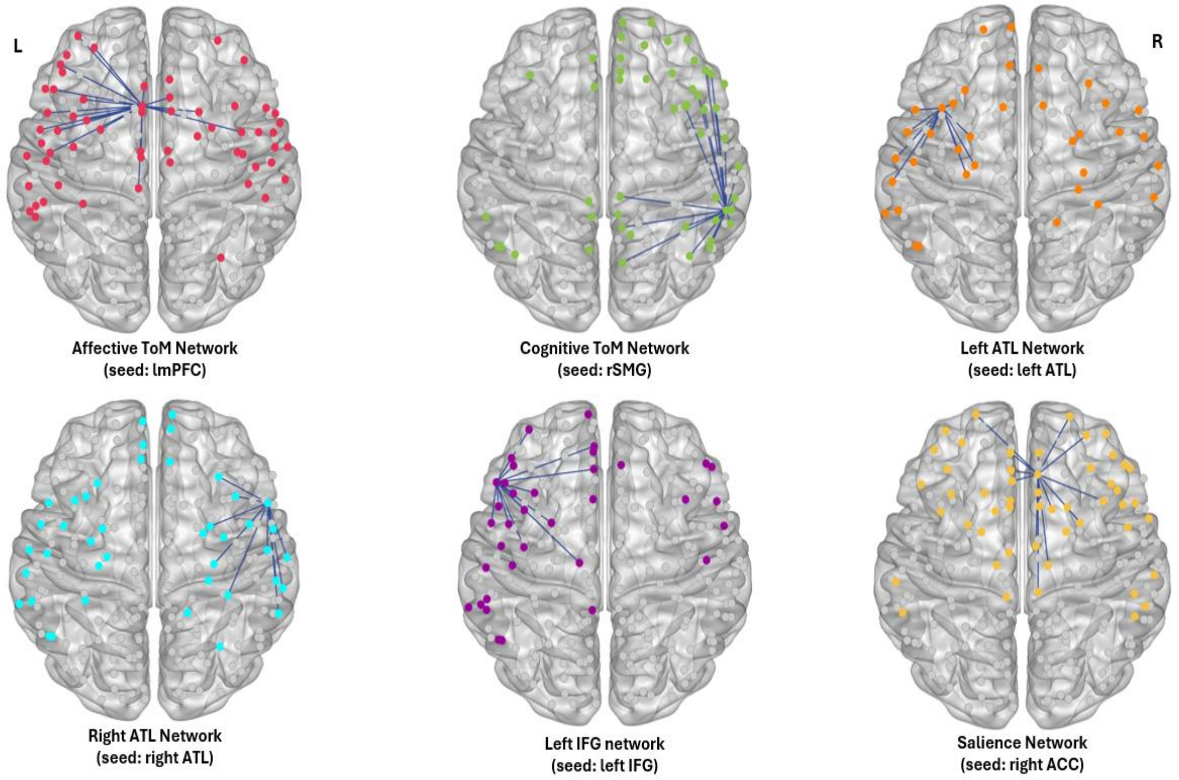

Each dot represents a node within the functional network. Colored lines indicate direct connections departing from the network seed in healthy controls. Please note that no statistical inference is made at the level of direct seed connections.

Abbreviations: ToM = Theory of Mind; lmPFC = left medial Prefrontal Cortex; rSMG = right Supramarginal Gyrus; ATL = Anterior Temporal Lobe; IFG = Inferior Frontal Gyrus; ACC = Anterior Cingulate Cortex.

Supplementary Figure 3. Between group differences in network-based regional connectivity and direct connections from seed within the left anterior temporal lobe (ATL) network.

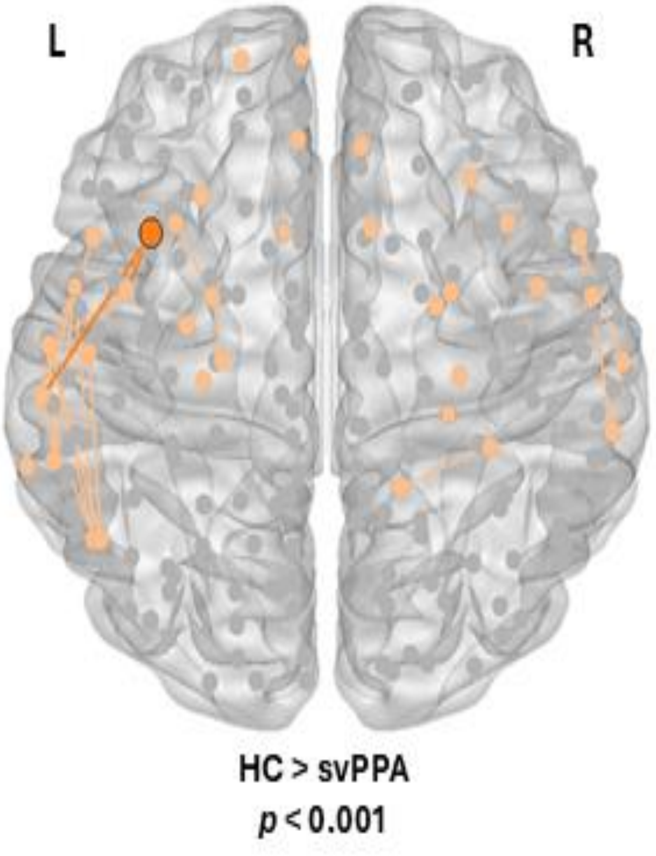

Connections show reduced functional connectivity (FC) in svPPA (N = 17) vs HC (N = 48). Edges were included in a network component if they showed a significant difference between groups (p < 0.05, ANCOVA). Component-level significance was determined via 5000 permutations, controlling for family-wise error (FWE).

Each dot represents a node within the functional network. Light-colored lines indicate all connections significantly altered within the identified network and for the given contrast, whereas darker lines highlight direct connections departing from the seed region (seed region for the left ATL Network = left anterior temporal lobe).

The reported p-values refer to significant effects at the network level and are permutation-corrected and adjusted for age, sex, and education. Please note that no statistical inference is made at the level of direct seed connections.

Abbreviations: HC = Healthy Controls; svPPA = semantic variant Primary Progressive Aphasia.

Supplementary Figure 4. **Between group differences in network-based regional connectivity and direct connections from seed within the right anterior temporal lobe (ATL) network.**

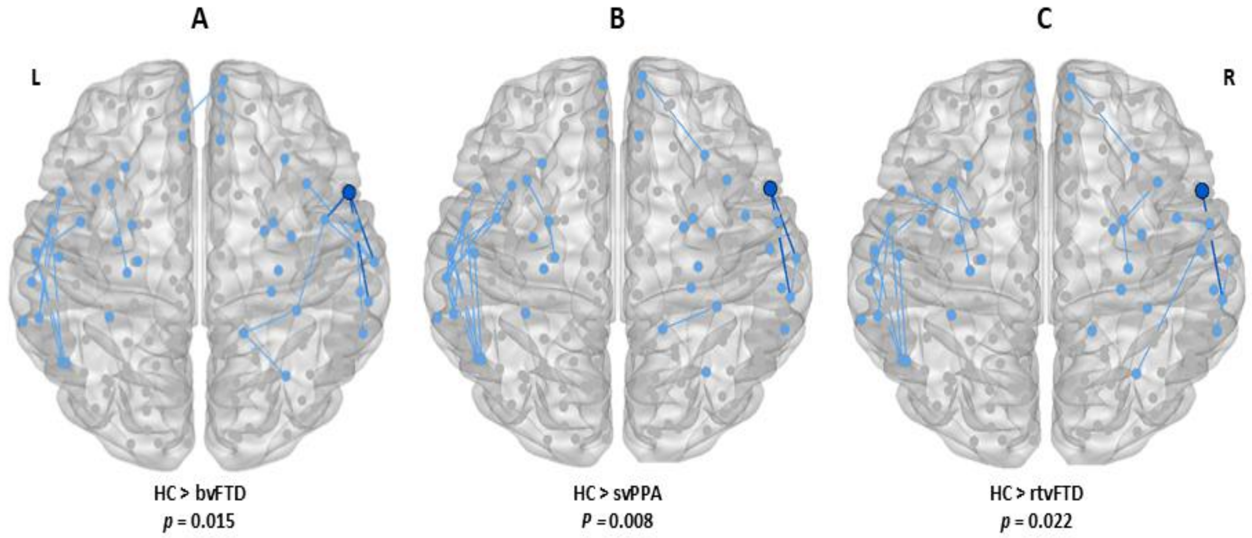

Connections show reduced functional connectivity (FC) across comparisons: bvFTD (N = 23) vs HC (N = 48; panel A), svPPA (N = 17) vs HC (panel B), rtvFTD (N = 13) vs HC (panel C). Edges were included in a network component if they showed a significant difference between groups ($p < 0.05$, ANCOVA). Component-level significance was determined via 5000 permutations, controlling for family-wise error (FWE).

Each dot represents a node within the functional network. Light-colored lines indicate all connections significantly altered within the identified network and for the given contrast, whereas darker lines highlight direct connections departing from the seed region (seed region for the right ATL Network = right anterior temporal lobe).

The reported p-values refer to significant effects at the network level and are permutation-corrected and adjusted for age, sex, and education. Please note that no statistical inference is made at the level of direct seed connections.

Abbreviations: HC = Healthy Controls; bvFTD = behavioral variant Frontotemporal Dementia; svPPA = semantic variant Primary Progressive Aphasia; rtvFTD = right temporal variant Frontotemporal Dementia.

Supplementary Figure 5. **Between group differences in network-based regional connectivity and direct connections from seed within the left inferior frontal gyrus (IFG) network.**

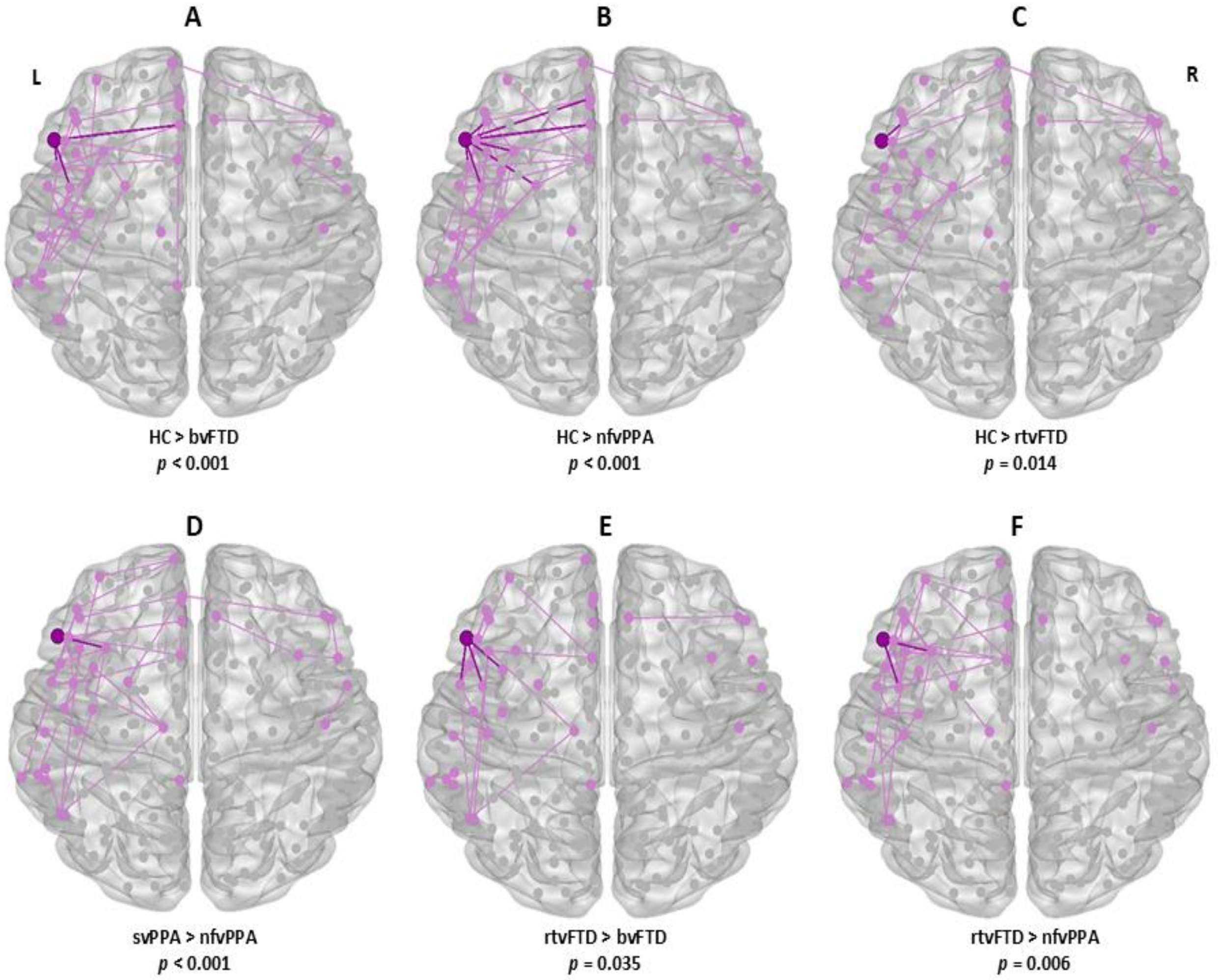

Connections show reduced functional connectivity (FC) across comparisons: bvFTD (N = 23) vs HC (N = 48; panel A), nfvPPA (N = 14) vs HC (panel B), rtvFTD (N = 13) vs HC (panel C), nfvPPA vs svPPA (N = 17; panel D), bvFTD vs rtvFTD (panel E), nfvPPA vs rtvFTD (panel F). Edges were included in a network component if they showed a significant difference between groups ($p < 0.05$, ANCOVA). Component-level significance was determined via 5000 permutations, controlling for family-wise error (FWE).

Each dot represents a node within the functional network. Light-colored lines indicate all connections significantly altered within the identified network and for the given contrast, whereas darker lines highlight direct connections departing from the seed region (seed region for the left IFG Network = left inferior frontal gyrus).

The reported p-values refer to significant effects at the network level and are permutation-corrected and adjusted for age, sex, and education. Please note that no statistical inference is made at the level of direct seed connections.

Abbreviations: HC = Healthy Controls; bvFTD = behavioral variant Frontotemporal Dementia; nfvPPA = nonfluent variant Primary Progressive Aphasia; svPPA = semantic variant Primary Progressive Aphasia; rtvFTD = right temporal variant Frontotemporal Dementia.

Supplementary Figure 6. Between group differences in network-based regional connectivity and direct connections from seed within the SN.

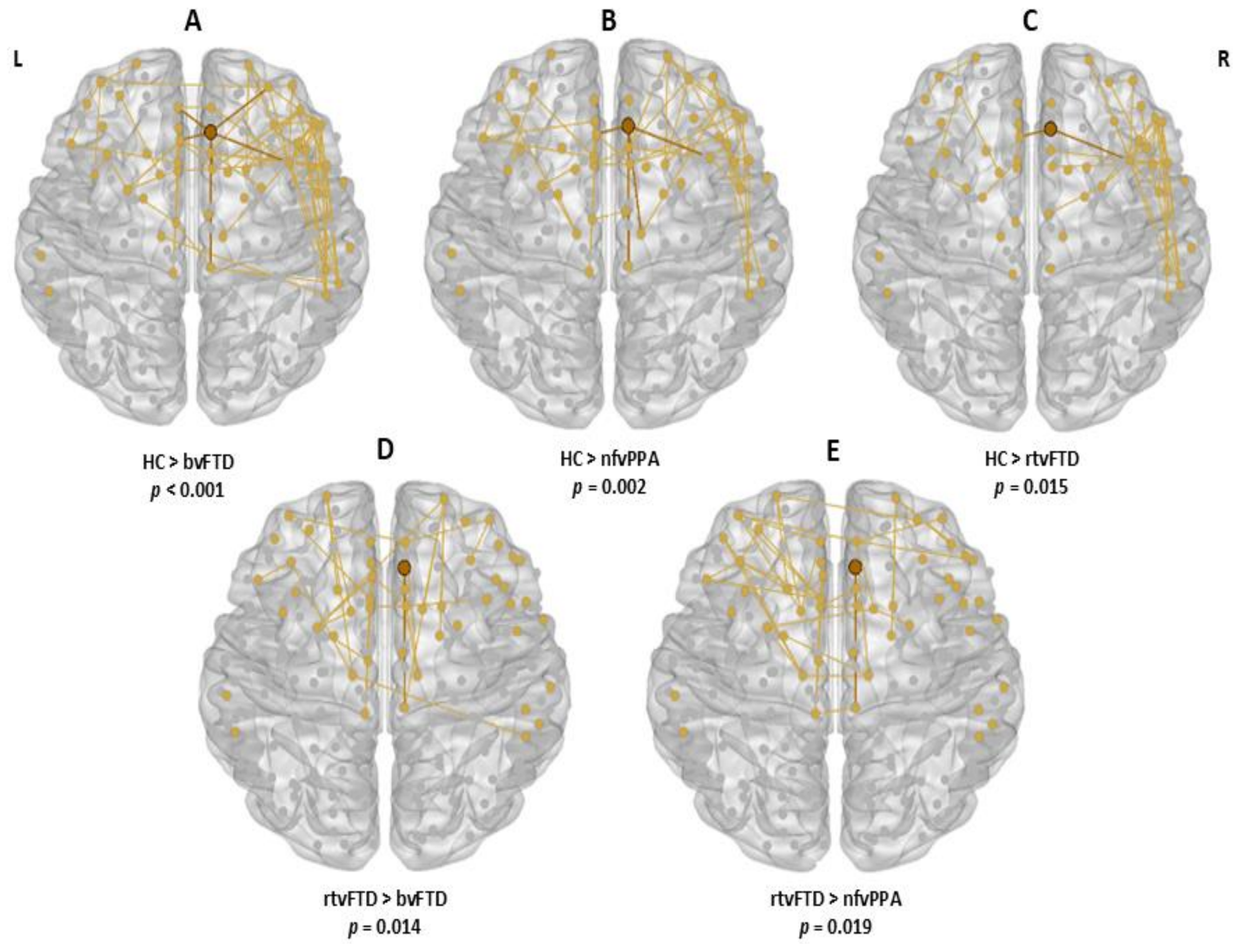

Connections show reduced functional connectivity (FC) across comparisons: bvFTD (N = 23) vs HC (N = 48; panel A), nfvPPA (N = 14) vs HC (panel B), rtvFTD (N = 13) vs HC (panel C), bvFTD vs rtvFTD (panel D), nfvPPA vs rtvFTD (panel E). Edges were included in a network component if they showed a significant difference between groups ($p < 0.05$, ANCOVA). Component-level significance was determined via 5000 permutations, controlling for family-wise error (FWE).

Each dot represents a node within the functional network. Light-colored lines indicate all connections significantly altered within the identified network and for the given contrast, whereas darker lines highlight direct connections departing from the seed region (seed region for the SN Network = right anterior cingulate cortex).

The reported p-values refer to significant effects at the network level and are permutation-corrected and adjusted for age, sex, and education. Please note that no statistical inference is made at the level of direct seed connections.

Abbreviations: HC = Healthy Controls; bvFTD = behavioral variant Frontotemporal Dementia; nfvPPA = nonfluent variant Primary Progressive Aphasia; rtvFTD = right temporal variant Frontotemporal Dementia.
